# An Alternative Nested Reading Frame May Participate in the Stress-Dependent Expression of a Plant Gene

**DOI:** 10.3389/fpls.2017.02137

**Published:** 2017-12-19

**Authors:** Ekaterina V. Sheshukova, Tatiana V. Komarova, Natalia M. Ershova, Anastasia V. Shindyapina, Yuri L. Dorokhov

**Affiliations:** ^1^Department of Genetics and Biotechnology, N.I. Vavilov Institute of General Genetics, Russian Academy of Sciences, Moscow, Russia; ^2^A.N. Belozersky Institute of Physico-Chemical Biology, Lomonosov Moscow State University, Moscow, Russia

**Keywords:** Kunitz peptidase inhibitor, matryoshka gene, alternative nested reading frame, abiotic and biotic stress, virus, bacteria, gene, expression

## Abstract

Although plants as sessile organisms are affected by a variety of stressors in the field, the stress factors for the above-ground and underground parts of the plant and their gene expression profiles are not the same. Here, we investigated *NbKPILP*, a gene encoding a new member of the ubiquitous, pathogenesis-related Kunitz peptidase inhibitor (KPI)-like protein family, that we discovered in the genome of *Nicotiana benthamiana* and other representatives of the *Solanaceae* family. The *NbKPILP* gene encodes a protein that has all the structural elements characteristic of KPI but in contrast to the proven *A. thaliana* KPI (AtKPI), it does not inhibit serine peptidases. Unlike roots, *NbKPILP* mRNA and its corresponding protein were not detected in intact leaves, but abiotic and biotic stressors drastically affected *NbKPILP* mRNA accumulation. In search of the causes of suppressed *NbKPILP* mRNA accumulation in leaves, we found that the *NbKPILP* gene is “matryoshka,” containing an alternative nested reading frame (ANRF) encoding a 53-amino acid (aa) polypeptide (53aa-ANRF) which has an amphipathic helix (AH). We confirmed ANRF expression experimentally. A vector containing a GFP-encoding sequence was inserted into the *NbKPILP* gene in frame with 53aa-ANRF, resulting in a 53aa-GFP fused protein that localized in the membrane fraction of cells. Using the 5′-RACE approach, we have shown that the expression of ANRF was not explained by the existence of a cryptic promoter within the *NbKPILP* gene but was controlled by the maternal *NbKPILP* mRNA. We found that insertion of mutations destroying the 53aa-ANRF AH resulted in more than a two-fold increase of the *NbKPILP* mRNA level. The *NbKPILP* gene represents the first example of ANRF functioning as a repressor of a maternal gene in an intact plant. We proposed a model where the stress influencing the translation initiation promotes the accumulation of NbKPILP and its mRNA in leaves.

## Introduction

Plants in natural field conditions are constantly exposed to a variety of abiotic and biotic environmental factors (Wang et al., 2003; Chaves and Oliveira, 2004; Nakashima and Yamaguchi-Shinozaki, 2006; Hirel et al., 2007; Bailey-Serres and Voesenek, 2008; Atkinson and Urwin, 2012; Suzuki et al., 2014; Ramegowda and Senthil-Kumar, 2015).

A set of stress factors and, accordingly, gene expression profiles for the underground and above-ground parts of plants differ (Freschet et al., 2015; Islam et al., 2015a,b; Liu et al., 2015; Chmielewska et al., 2016). Wind, rain, and herbivorous insects cause mechanical damages mainly to the above-ground part of the plants (Jaffe and Forbes, 1993; Sampathkumar et al., 2014; Savatin et al., 2014; Lup et al., 2016), which increase opportunities for penetration by bacteria and viruses (Carr et al., 2010; Ryu, 2015). Plant roots suffer primarily from soil bacteria (Alegria Terrazas et al., 2016), root-feeding herbivores (Kergunteuil et al., 2016), and salinity (Hanin et al., 2016). Cold, lack of water or lack of nutrients have a generalized effect on the plant (Suzuki et al., 2014; Ramegowda and Senthil-Kumar, 2015).

The plant, in response to the impact of a stress factor, switches on protective mechanisms, including generalized cellular mobilization and accumulation of mRNAs that direct the synthesis of protective proteins in cells (Dowen et al., 2012; Zavaliev et al., 2013; Crisp et al., 2016; Yang et al., 2017; Zhang and Sonnewald, 2017). Termination of the stress-factor impact cancels the synthesis of protective protein mRNAs and is followed by their degradation. Such repeated effects create a kind of “memory” (Crisp et al., 2016), which allows plants to shorten the time for a protective response. The reaction of the plant genome to the stress is carried out in close interaction with the plastid and mitochondrial genomes via anterograde and retrograde signaling (Leister et al., 2017). A main phytohormone, abscisic acid, plays an essential role in modifying the expression levels of stress-responsive genes (Baek et al., 2017; Vishwakarma et al., 2017). Stress-dependent modification of gene expression can be regulated through transcriptional factors (Eulgem and Somssich, 2007; Jang, 2016; Baek et al., 2017; Fang et al., 2017) and post-transcriptional mechanisms including alternative splicing (Shang et al., 2017) and mRNA nucleocytoplasmic trafficking via the nuclear pores (Yang et al., 2017). The translation of mRNA is also a mechanism for responding to stress, especially the stages involving the 5′ terminal oligopyrimidine tract and translation initiation factors eIF4E and eIF2a (Sesma et al., 2017).

It is known that the same genes related to defense provide a different level of mRNA accumulation in leaves and roots after challenge with abiotic factors (De Nardi et al., 2006; Bazakos et al., 2015; Corso et al., 2015; Freschet et al., 2015; Liu et al., 2015; Pucholt et al., 2015; Chmielewska et al., 2016; Fan et al., 2016; Goralski et al., 2016; Gurung et al., 2016; Miao et al., 2016; Nguyen et al., 2016; Kobayashi et al., 2017). Similar leaf-root differences after viral (Sohal et al., 1999; Germundsson et al., 2002; Andika et al., 2016), fungal (Galiana et al., 1997; Okubara and Paulitz, 2005), bacterial (Zdor and Anderson, 1992; de A Gerhardt et al., 1997; Gruau et al., 2015), and insect attack (Hermsmeier et al., 2001) were noted for various genes such as pathogenesis-related (PR) genes (Fu and Dong, 2013; Parmar et al., 2017) and genes encoding peptidase inhibitors (PIs) (Hörger and van der Hoorn, 2013; Jashni et al., 2015; Grosse-Holz and van der Hoorn, 2016; Misas-Villamil et al., 2016). PIs are classified into superfamilies which are subcategorized further into specific families (Rawlings et al., 2014, 2016). Among them is the Kunitz peptidase inhibitor (KPI) family belonging to the serine PI group (https://www.ebi.ac.uk/merops), which includes proteins of ~20 kD with one or two disulfide bonds and a single reactive site (Oliva et al., 2010, 2011; Santamaría et al., 2014; Guo et al., 2015). Functional analysis of the poplar KPI family revealed biochemical diversity and multiplicity in defense reactions (Major and Constabel, 2008). Moreover, a study of white clover has shown that some members of the KPI family are expressed only in roots and not in leaves or other parts of plants (Islam et al., 2015a,b).

Until recently, stress response studies and leaf-root differences in mRNA accumulation studies focused primarily on the mechanisms of transcriptional promoter involvement in gene regulation, activation of transcription factors, and chromatin cytosine methylation in the promoter region of certain genes to prevent the access of transcriptional activators (Komarnytsky and Borisjuk, 2003; Jones and Dangl, 2006; Ryu, 2015; Espinas et al., 2016; Neto et al., 2016; Jiang et al., 2017; Marand et al., 2017). Explanations of the differences in the expression of a gene in leaves and roots are usually based on mechanisms involving transcription factors (Kobayashi et al., 2017), hormones (Fujita et al., 2006), volatile organic compounds (Duran-Flores and Heil, 2016), and mobile RNA transcripts (Saplaoura and Kragler, 2016).

Now it has become clear that the leaf-root differences in gene expression and their reactions to stress cannot be explained by only the regulation of gene transcription. So far, no studies have been conducted on alternative nested reading frames (ANRF) in mRNA and their role in mRNA stability to explain the pattern of certain gene expression levels in leaf and root tissues. Although until recently there was an established opinion that one RNA transcript encodes a single protein, the latest data from proteogenomics pointed to the existence of an exception to this rule, which, in many respects, changes the customary meaning of the term “gene” (Kochetov et al., 2013; Liu and Qian, 2014; Mouilleron et al., 2016). The coding potential of such ANRF was not taken into account in most cases, primarily due to the lack of information on the possibility of their translation to polypeptides in a cell (Andrews and Rothnagel, 2014). Recent discoveries of the cell polypeptides encoded by ANRF created the concept of a gene system which could be described as a “matryoshka” (Ribrioux et al., 2008). Improvement of research techniques led to the detection of double-coding transcripts in mammals, such as the *TRP-1* gene transcript encoding the TYRP1 tumor antigen (Wang et al., 1996), the *caspase 1* (*CASP1* or *ICE*) transcript (Ronsin et al., 1999), the prion protein gene *PRNP* (Vanderperre et al., 2011, 2013), and the gene encoding Ataxin-1 (ATXN1) (Bergeron et al., 2013).

In principle, besides the ANRF in the 5′-leader sequence region designated as upstream open reading frames (uORFs), plant mRNAs contain a plurality of ANRF in the main open reading frame (Hayden and Jorgensen, 2007; Tran et al., 2008; Vaughn et al., 2012). The role of uORF expression in plant stress response was confirmed by ribosomal profiling in normal and stress conditions (Juntawong et al., 2014; Tanaka et al., 2016; Bailey-Serres and Ma, 2017; Schepetilnikov and Ryabova, 2017; Sesma et al., 2017; Xu et al., 2017a,b). Expression of ANRF located in the coding portion of the main gene has only been demonstrated for the maize gene *Zm908* (Dong et al., 2013; Hanada et al., 2013).

Here, we identified and described the properties of a novel *Nicotiana benthamiana* gene encoding a KPI-like protein (NbKPILP). Unlike roots, *NbKPILP* mRNA and its corresponding protein were not detected in intact leaves, but prolonged darkness and bacterial or viral infection stimulated its mRNA accumulation. We found that the *NbKPILP* gene is common for *Solanaceae* plants and belongs to a matryoshka gene family containing an ANRF that encodes a 53-amino acid (aa) polypeptide (53aa-ANRF) which contains an amphipathic helix (AH). Our experimental approaches allowed identification of the 53aa-ANRF that affected the level of *NbKPILP* mRNA accumulation in intact leaves. The existence of a cryptic promoter within the *NbKPILP* gene was excluded using the 5′-RACE approach. We found that insertion of mutations destroying the 53aa-ANRF AH resulted in an increase of *NbKPILP* mRNA accumulation. The *NbKPILP* gene is the first example of an ANRF influencing maternal mRNA accumulation in leaves.

## Materials and methods

### Plant growth conditions

*Nicotiana benthamiana* plants were grown in soil in a controlled environment under a 16/8 h day/night cycle.

### Plasmid and vectors

To create 35S-NbKPILP construct NbKPILP-encoding sequence was obtained by PCR using “NbKPILP(KpnI)d” and “NbKPILP(SalI)r” primers and *N. benthamiana* total cDNA as a template. PCR product was subsequently digested with KpnI and SalI and inserted into pCambia1300-based binary vector containing *Cauliflower mosaic virus* (CaMV) 35S promoter and 35S terminator of transcription (pCambia-35S) via KpnI/SalI sites. To create 35S-AtKPI construct AtKPI-encoding sequence was obtained by PCR using “AtKPI(SacI)d” and “AtKPI(PstI)r” primers and total *A. thaliana* cDNA as a template. PCR product was digested with SacI and PstI and inserted into pCambia-35S SacI/PstI sites.

To obtain the (SS-)NbKPILP-6xHis plasmid, SacI, and HindIII sites were introduced at the 5′- and the 3′-ends of (SS-)NbKPILP-encoding sequence, respectively, through PCR with primers “ss-NbKPILP(SacI)d” and “NbKPILP(HindIII)r.” The (SS-)NbKPILP fragment flanked with SacI and HindIII was cloned into pQE30 (QIAGEN, Holland) plasmid digested with SacI and HindIII, to generate the (SS-)NbKPILP-6xHis construct. To obtain the (SS-)AtKPI-6xHis construct (SS-)AtKPI sequence was amplified using the primer pair “AtKPI(BamHI)d” and “AtKPI(HindIII)r,” PCR product was subsequently digested with BamHI and HindIII endonucleases. That fragment was cloned into pQE30 (QIAGEN, Holland) plasmid digested with BamHI and HindIII.

For the 35S-NbKPILP(53aa-GFP) and 35S-(SS-) NbKPILP(53aa-GFP) constructs the fragments, containing 1–565 or 70–565 nt of *NbKPILP* ORF, respectively, were amplified using the following pairs of primers: “NbKPILP(KpnI)d”/“53aa_end(BamHI)r” or “ss-NbKPILP(SacI)d”/“53aa_end(BamHI)r.” The PCR products were digested with KpnI/BamHI and together with the fragment containing *GFP* flanked with BamHI and PstI were inserted into digested with KpnI and PstI pCampia-35S resulting in 35S-NbKPILP(53aa-GFP) and 35S-(SS-)NbKPILP(53aa-GFP) constructs, respectively.

The 35S-NbKPILP(ACG) construct was created in several steps. First, site-directed mutagenesis using “NbKPILP(KpnI)d”/“53aa(ACG)r” and “53aa(ACG)d”/“NbKPILP(SalI)r” pairs of primers and 35S-NbKPILP plasmid as a template was performed. Second, the final PCR product, containing NbKPILP(ACG) sequence was obtained using overlap PCR approach with “NbKPILP(KpnI)d” and “NbKPILP(SalI)r” pair of primers. The product of overlap PCR was digested with KpnI and SalI and inserted into pCambia-35S via KpnI/SalI sites.

The crTMV-53aa and crTMV-53aa_mut vectors were created in several steps. First, a PCR fragment containing 53aa-ANRF-encoding sequence (407–568 nt of NbKPILP ORF) was obtained using “53aa(NcoI)d” and “53aa(XhoI)r” pair of primers. For substituted variant (53aa_mut) the PCR fragment was obtained using “53aa(NcoI)d”/“53aa(AHmut)r” and “53aa(AHmut)d”/“53aa(XhoI)r” pairs of primers for site directed mutagenesis and “53aa(NcoI)d”/“53aa(XhoI)r” pair of primers for overlap PCR. The resulting PCR products, encoding 53aa-ANRF or 53aa_mut-ANRF, were digested with NcoI and XhoI and inserted into plasmid IC-2 (Komarova et al., 2011), containing 3′-region of crTMV movement protein (MP) gene, 3′-UTR of crTMV and *nos* transcription terminator. The resulting constructs were digested with EcoRI/XhoI and a fragment containing part of *MP*, 53aa-ANRF (or 53aa_mut-ANRF), crTMV 3′-UTR and *nos* terminator was inserted into TMV-HC vector (Komarova et al., 2011) via EcoRI/XhoI sites resulting in crTMV-53aa or crTMV-53aa_mut construct, respectively.

To obtain the plasmid encoding NbKPILP(53aa) fused to the DYKDDDDKDYKDVDDYKDDDDK (3xFLAG) sequence (Ueda et al., 2011), a fragment containing full *NbKPILP* sequence without stop codon was amplified using “NbKPILP(KpnI)d” and “NbKPILP(XbaI)r” primers and subsequently digested with KpnI and XbaI. The 3xFLAG encoding sequence was generated annealing the primers “3xFlag(XbaI)d” and “3xFlag(SalI)r,” resulting in a fragment with overhangs corresponding to XbaI and SalI “sticky” ends. These fragments were inserted into pCambia-35S via KpnI/SalI sites to generate the 35S-NbKPILP(53aa):3xFLAG plasmid. For 35S-NbKPILP(53aa_mut):3xFLAG construct, fragment containing NbKPILP(53aa_mut) was obtained by site-directed mutagenesis using primer pairs “NbKPILP(KpnI)d”/“53aa(AHmut)r” and “53aa(AHmut)d”/“NbKPILP(XbaI)r” and 35S-NbKPILP as a template. Then these PCR fragments were used in overlap PCR with “NbKPILP(KpnI)d”/“NbKPILP(XbaI)r” primers. The resulting product of overlap PCR was digested with KpnI and XbaI and inserted into pCambia-35S together with 3xFLAG-encoding fragment flanked with XbaI and SalI resulting in 35S-NbKPILP(53aa_mut):3xFLAG.

A full list of the oligonucleotides used for cloning is presented in Table S1.

### Transcription start site determination using a step-out rapid amplification of cDNA 5′-end (5′-race) approach

The 5′-RACE of *NbKPILP* cDNA was performed using the Mint RACE cDNA amplification set (Evrogen, Russia) according to manufacturer's instructions. The following gene-specific primers were used: “pr1;” “pr2” (Table S1).

### Agroinjection experiments

The *Agrobacterium tumefaciens* strain GV3101 was transformed with individual binary constructs and grown at in LB medium supplemented with 50 mg/l rifampicin, 25 mg/l gentamycin, and 50 mg/l kanamycin. The *Agrobacterium* from an overnight culture were resuspended in 10 mM MES buffer (pH 5.5) supplemented with 10 mM MgSO_4_ and adjusted to a final OD_600_ of 0.1. Agroinjection was performed using nearly fully expanded *N. benthamiana* leaves attached to the intact plant. A bacterial suspension was infiltrated into the leaf tissue using a 2-ml syringe, after which the plants were grown under greenhouse conditions at and a 16/8 h light/dark photoperiod unless otherwise specified.

### *N. tabacum* inoculation with TMV

Lower leaves of *N. tabacum* cv. Samsun plants were mechanically inoculated with TMV virions (100 mg/ml) in 50 mM sodium phosphate buffer, pH 7.0, in the presence of Celite, as described previously (Dorokhov et al., 1981).

### GFP visualization

GFP fluorescence in the inoculated leaves was monitored by illumination with a handheld UV source, λ = 366 nm. To visualize 53aa-GFP subcellular localization, imaging was performed 72 h after agroinfiltration with 35S-*NbKPILP(53aa-GFP)* in the presence of a vector encoding silencing suppressor P19 from *Tomato bushy stunt virus* using an LSM510 confocal laser scanning microscope (Zeiss, Germany). Excitation wavelength was 488 nm and detection window was 493–550 nm.

### Western blot analysis

For Western blot analysis, the proteins from agroinjected leaves were divided into S17, P17, P1, and CW crude fractions according to Deom et al. (1990) with modifications. Briefly, frozen plant material was ground to a powder in liquid nitrogen followed by addition of three volumes of ice-cold-modified GB buffer (100 mM Tris, pH 8.0, 0.4 M sucrose, 10 mM KCL, 5 mM MgCl_2_, 10 mM β-mercaptoethanol). The obtained slurry was filtered through a double-layered Miracloth (Millipore/Merck). The material retained on the filter was collected and washed (30–60 min incubation followed by centrifugation at 1,000 × *g*) 5–8 times with GB-buffer supplemented with 0.1% Triton X-100 (the final wash was performed without Triton X-100) to obtain the CW-enriched fraction. The filtrate was centrifuged at 1,000 × *g* for 10 min to obtain the P1 (pellet enriched with nuclei) fraction. Supernatant was further divided into S17 (supernatant enriched with soluble proteins) and P17 (pellet enriched with membrane non-soluble proteins) fractions after centrifugation at 17,000 × *g* for 30 min. Pellets from CW, P1 and P17 fractions were resuspended in one volume of 1xPBS.

Total protein was extracted from plant tissues using TriReagent (MRC) according to the manufacturer's instructions.

Aliquotes from all fractions were analyzed through SDS-polyacrylamide gel electrophoresis and blotted onto polyvinylidene difluoride membranes (GE Healthcare). For GFP or NbKPILP detection, the membranes were probed with corresponding antibodies: goat anti-GFP antibodies conjugated with horseradish peroxidase (Rockland Immunochemicals) or mouse polyclonal antibodies against recombinant NbKPILPx6His. Anti-mouse antibodies conjugated with horseradish peroxidase (Rockland Immunochemicals) were used as secondary antibodies. The bands were visualized using the chemiluminescence ECL kit (GE Healthcare).

### Q-PCR analysis of transcript concentrations

Total RNA was extracted from plant tissues using TriReagent (MRC) according to the manufacturer's instructions. The RNA concentration was determined using a Nanodrop ND-1000 spectrophotometer (Isogen Life Sciences). All RNA samples had a 260:280 absorbance ratio between 1.9 and 2.1. The synthesis of the first strand, followed by real-time qPCR, was performed as described in Dorokhov et al. (2012). Briefly, 0.1 mg of random hexamers and 0.1 mg of oligo-dT primer were added to 2 mg of total RNA to obtain cDNA through reverse transcription using Superscript II reverse-transcriptase (Invitrogen), according to the manufacturer's protocol. Real-time quantitative PCR was carried out using the iCycler iQ real-time PCR detection system (Bio-Rad). Target genes were detected using sequence-specific primers (Table S2) and Eva Green master mix (Syntol) according to the manufacturer's instructions. Each sample was run in triplicate, and a non-template control was added to each run. A minimum of five biological replicates were performed.

### Statistics

Student's *t*-test was performed using Excel (Microsoft, Redmond, WA). *P* < 0.05 were considered significant.

## Results

### Identification and analysis of the *N. benthamiana* gene encoding KPI-like protein (NbKPILP)

**Figure 1:**
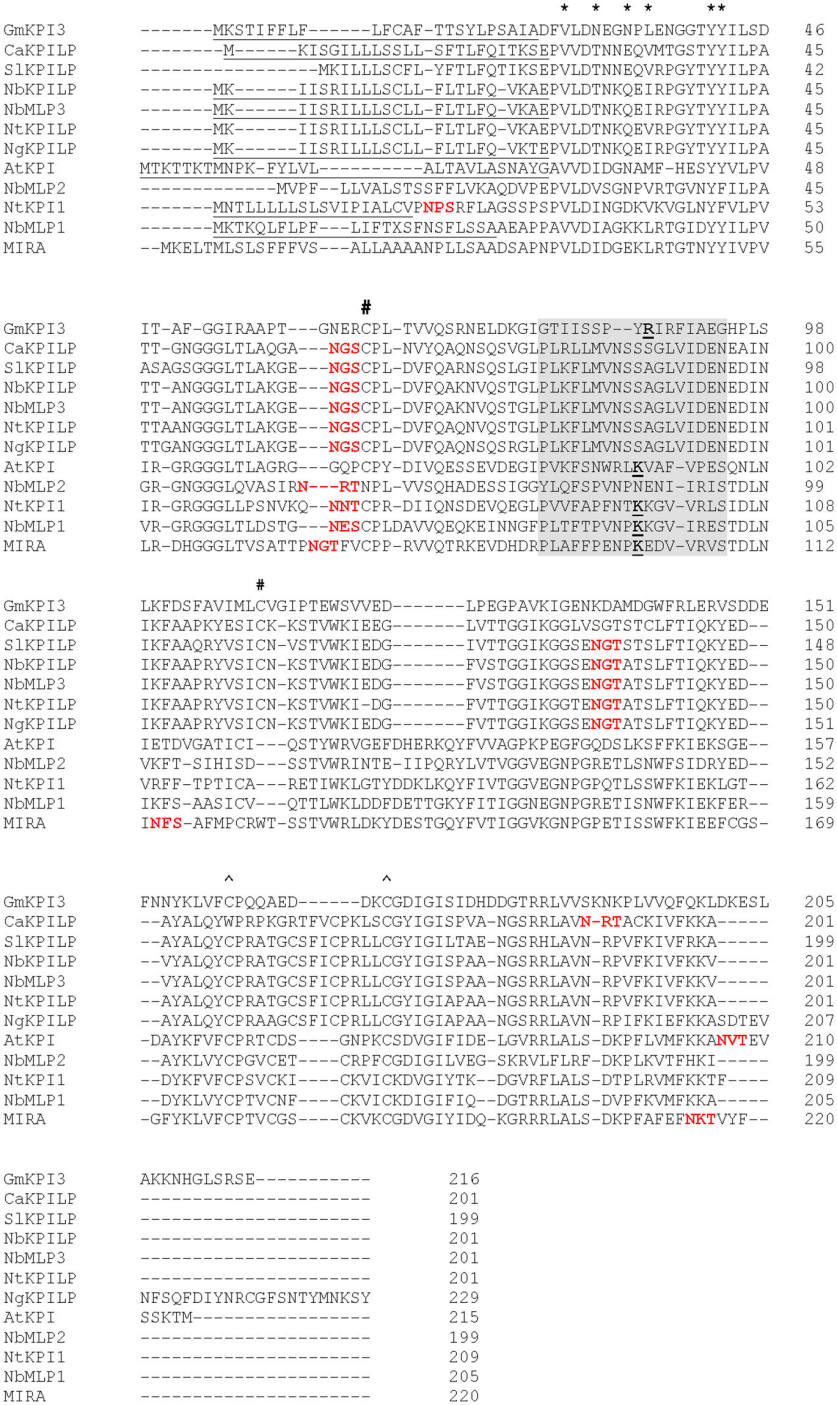
Amino acid sequence alignment of NbKPILP with predicted amino acid sequences of other homologous proteins. The alignment was performed using the CLUSTAL programme (http://www.ebi.ac.uk/Tools/msa/clustalo). KPI-like proteins, CaKPILP (*Capsicum annuum* KPILP, sequence ID Ntab-BX_AWOK-SS1956), *N. benthamiana* miraculin-like proteins (NbMLP1, 2, and 3) (Goodwin et al., 2012) and NtKPILP (*N. tabacum* KPILP, sequence ID Ntab-BX_AWOK-SS1956) were retrieved from the Sol Genomics Network (http://solgenomics.net/). SlKPILP, *Solanum lycopersicum* KPI-like protein (accession number K4BJT7) and NgKPILP, *N. glutinosa* biotic cell death-associated protein (accession number Q850R9) were retrieved from the NCBI database. GmKPI3, *Glycine max* KPI encoded by the *KTI3* gene (NCBI accession number P01070) (De Meester et al., 1998), *N. tabacum* KPI1 (NCBI accession number B8Y888) (Huang et al., 2010), AtKPI, *Arabidopsis thaliana* KPI (NCBI accession number Q8RXD5) (Li et al., 2008), *Richadella dulcifica/Synsepalum dulcificum* miraculin (MIRA) (NCBI accession number P13087). Signal sequences are underlined. The known structural features of GmKPI are annotated as follows: asterisks (^*^) above the alignment denote the Kunitz motif, # and ^∧^ signs denote pairs of Cys residues forming disulfide bonds, the reactive loop sequence is highlighted in gray, the P1 reactive site residues responsible for trypsin inhibition activity according to De Meester et al. (1998), Li et al. (2008) and Huang et al. (2010) and the PeptideCutter (http://web.expasy.org/peptide_cutter/) prediction are bolded and underlined. The NetNGlyc 1.0 server (http://www.cbs.dtu.dk/services/NetNGlyc/) predicted N-glycosylated sites are marked with bolded red text.

In a study of the transcriptome of *N. benthamiana* leaves infected with *Tobacco mosaic virus* (TMV), we detected an increased accumulation of mRNA (EMBL ID FN687760) encoding KPI-like protein (NbKPILP) (NCBI accession number D4IHB9), which according to the MEROPS classification (http://merops.sanger.ac.uk; Rawlings et al., 2014, 2016) belongs to the I3 family (clan IC), of which the soybean Kunitz trypsin inhibitor (*Glycine max*) is a typical member. To determine if there are any introns present within the *NbKPILP* ORF, the *N. benthamiana* genomic DNA and primers designed for the cDNA amplification were used to amplify the corresponding fragment. The amplified fragment length was 606-bp for both genomic DNA and cDNA. The genomic fragment sequence perfectly matched the cDNA-derived fragment (data not shown), which indicated the absence of an intron in the ORF-encoding part of chromosomal *NbKPILP* gene. Thus, like other plant KPIs (Jofuku and Goldberg, 1989; Ishikawa et al., 1994; Saarikoski et al., 1996; Gruden et al., 1997; Ashida et al., 2000; Tian et al., 2007; Huang et al., 2010; Bhattacharjee et al., 2014; Bunyatang et al., 2016), the *NbKPILP* gene does not contain introns, as shown by the chromosomal DNA sequence that we identified (data not shown) and a sequence search in the Sol Genomics Network (http://solgenomics.net/) and the *N. benthamiana* Genome and Transcriptome database (http://benthgenome.qut.edu.au/). The predicted amino acid sequence has high homology to KPILP of other *Solanaceae* species, including not only the earlier described *N. glutinosa* biotic cell death-associated protein (NgCDM1; Suh et al., 2003) but also KPILP sequences from tobacco, potato, tomato, and pepper (Figure 1). NbKPILP contains a predicted 24-aa signal sequence and has homology to members of the I3 family such as soybean KTI which possesses inhibitor activity against serine peptidase and is encoded by the *KTI3* gene (GmKTI3) (29.17% identity; Jofuku and Goldberg, 1989), *N. tabacum* KPI1 (Huang et al., 2010), and the *A. thaliana* KPI (EMBL ID Q8RXD5) (33.3% identity; Li et al., 2008). Figure 1 also shows alignment of NbKPILP with other sequences from the *N. benthamiana* genome: genes encoding miraculin-like protein 1, NbMLP1 (39.47% identity), NbMLP2 (35.75% identity), and NbMLP3, which is completely identical to NbKPILP (100.00% identity; Goodwin et al., 2012). Using the MEGA7 software (http://www.megasoftware.net/), a phylogenetic tree including KPIs and KPILPs with highest sequence identity to NbKPILP, was constructed (Figure S1). Inspection of the phylogenetic tree revealed that NbKPILP belongs to the same branch as other KPILPs from *Solanaceae* plants.

The NbKPILP likely undergoes glycosylation similar to *Synsepalum dulcificum* miraculin (MIRA) (Takahashi et al., 1990) which shows the presence of N-linked glycans at the Asn-71 and Asn-215 positions (the numbering includes signal sequences) (Figure 1). NbKPILP has putative N-glycosylation sites in Asn-60, Asn-86, and Asn-136 (Figure 1) according to a prediction by the NetNGlyc programme (http://www.cbs.dtu.dk/services/NetNGlyc/).

The NbKPILP has main sequence signatures of the KPI family (Major and Constabel, 2008; Huang et al., 2010; Guo et al., 2015) such as (i) the Kunitz motif, (ii) four cysteine residues arranged into two intra-chain disulfide bridges, and (iii) the reactive loop. However, there are significant differences in comparison with GmKPI3, AtKPI, or NbMLP1, for example (Figure 1). NbKPILP contains a lysine residue instead of glycine in the Kunitz motif and substitutions in the P1 reactive site that determines substrate specificity (Ser instead of Arg or Lys and Ala instead of Ile/Val; Goodwin et al., 2012; Guo et al., 2015). The reactive loop of NbKPILP, according to a prediction by PeptideCutter (http://www.expasy.org/tools/peptidecutter/), does not contain a P1 reactive site residue that interacts with trypsin, such as Arg63-Ile64 in the Kunitz-type soybean trypsin inhibitor (De Meester et al., 1998). Thus, NbKPILP is unlikely to have trypsin inhibitory activity. To test this prediction, we obtained a recombinant *E. coli* strain producing NbKPILP containing 6xHis tag and lacking the signal sequence [(SS-)NbKPILP-6xHis)]. (SS-)AtKPI-6xHis was used as a positive control with experimentally proven KPI activity *in vitro* (Li et al., 2008). Two substrates were used to evaluate the inhibitory activity of proteins: (i) p-nitroaniline, a synthetic substrate of trypsin which is characterized by high specificity of trypsin/substrate interaction and (ii) azocasein, a natural substrate of trypsin. (SS-)AtKPI-6xHis demonstrated a 90% decrease in protease activity with the synthetic substrate, and a 55% reduction with the natural substrate whereas (SS-) NbKPILP-6xHis did not show any trypsin inhibition on either substrate *in vitro* (data not shown).

### *NbKPILP* mRNA accumulation in *N. benthamiana* leaves is increased after incubation in darkness and foreign protein overproduction

**Figure 2:**
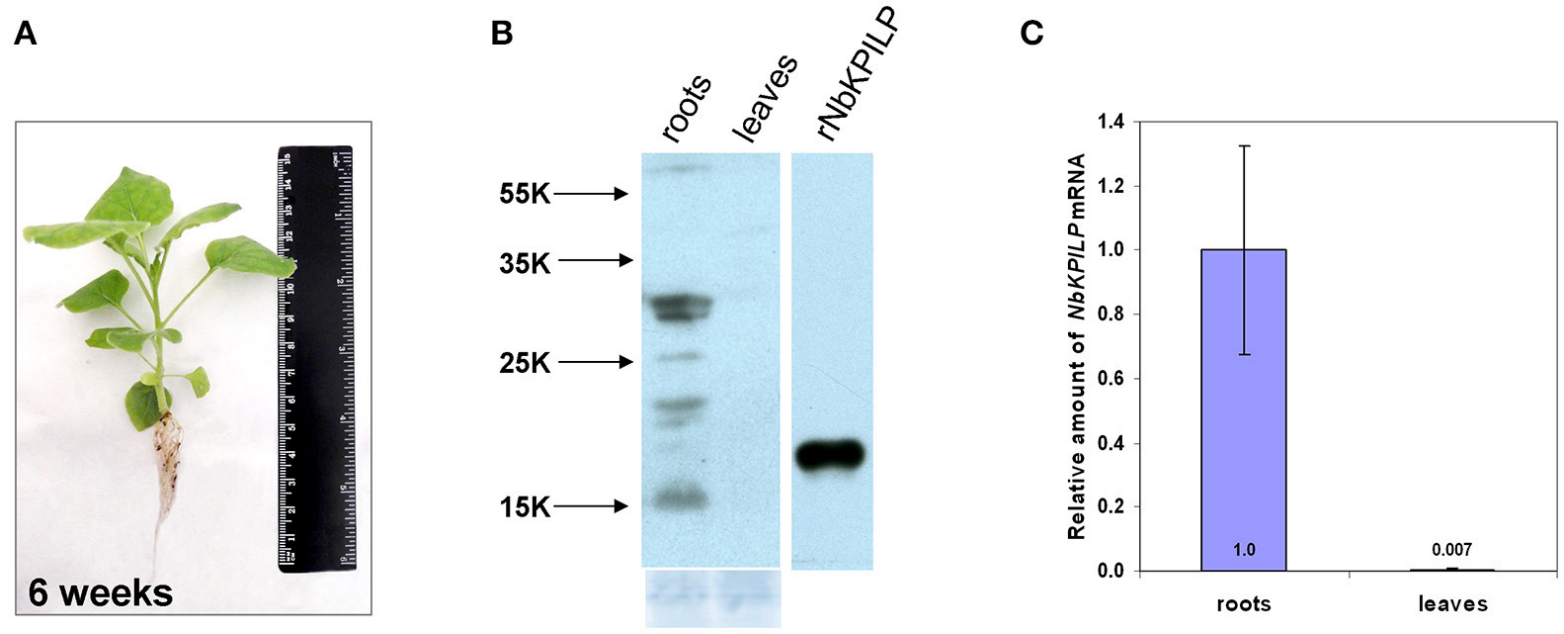
*NbKPILP* expression was observed in the roots but not in intact leaves. **(A)** View of a typical 6-week-old *N. benthamiana* plant used to analyse the content of RNA and protein. **(B)** Western blot analysis with *NbKPILP*-specific antibodies of total protein extracts from leaves and roots. The lower panel shows the protein loading control stained with Amido Black. rNbKPILP, recombinant NbKPILP with 6xHis-tag produced in *E. coli*. **(C)** Relative amounts of *NbKPILP* mRNA in roots and leaves quantified by qRT-PCR.

Biologically, the expression of KPI and KPILP in an intact plant varies widely in roots and leaves (Huang et al., 2010; Goodwin et al., 2012). We examined adult plants of *N. benthamiana* (Figure 2A) and did not identify NbKPILP in leaves using Western blot analysis (Figure 2B). Conversely, the protein analysis of the roots detected a set of protein bands, among which there was a major double band corresponding to proteins of 30 and 29 kDa, which significantly exceeds the predicted weight of a mature NbKPILP (19 kDa). Subcellular root fractionation showed the presence of NbKPILP mainly in the membrane fraction (data not shown), which is consistent with the presence of a signal sequence in the protein (Figure 1). It can be assumed that the significant differences in the content of NbKPILP in leaves and roots may occur due to differences in the level of accumulation of *NbKPILP* mRNA. We explored the *NbKPILP* mRNA levels in roots and mature leaves of healthy intact plants and showed that *NbKPILP* mRNA content in leaves was negligible, while in the roots it was almost three orders higher (Figure 2). The *N. benthamiana* Gene Expression Atlas programme (http://sefapps02.qut.edu.au/atlas/tREX6.php) confirmed our observation and also showed an increased expression of the *NbKPILP* gene in flowers (Figure S2A).

We analyzed the *NbKPILP* mRNA accumulation in the leaves and roots of *N. benthamiana* plants of different ages, starting from 2-week-old seedlings to 20-week-old flowering plants (Figure S2B). We observed that the *NbKPILP* mRNA content in leaves was low and independent of plant age, while the *NbKPILP* mRNA content in roots increased with plant age and was highest at the flowering stage (Figure S2C).

Thus, the intact *N. benthamiana* roots and leaves are radically different in NbKPILP content due to differences in the level of *NbKPILP* mRNA accumulation.

**Figure 3:**
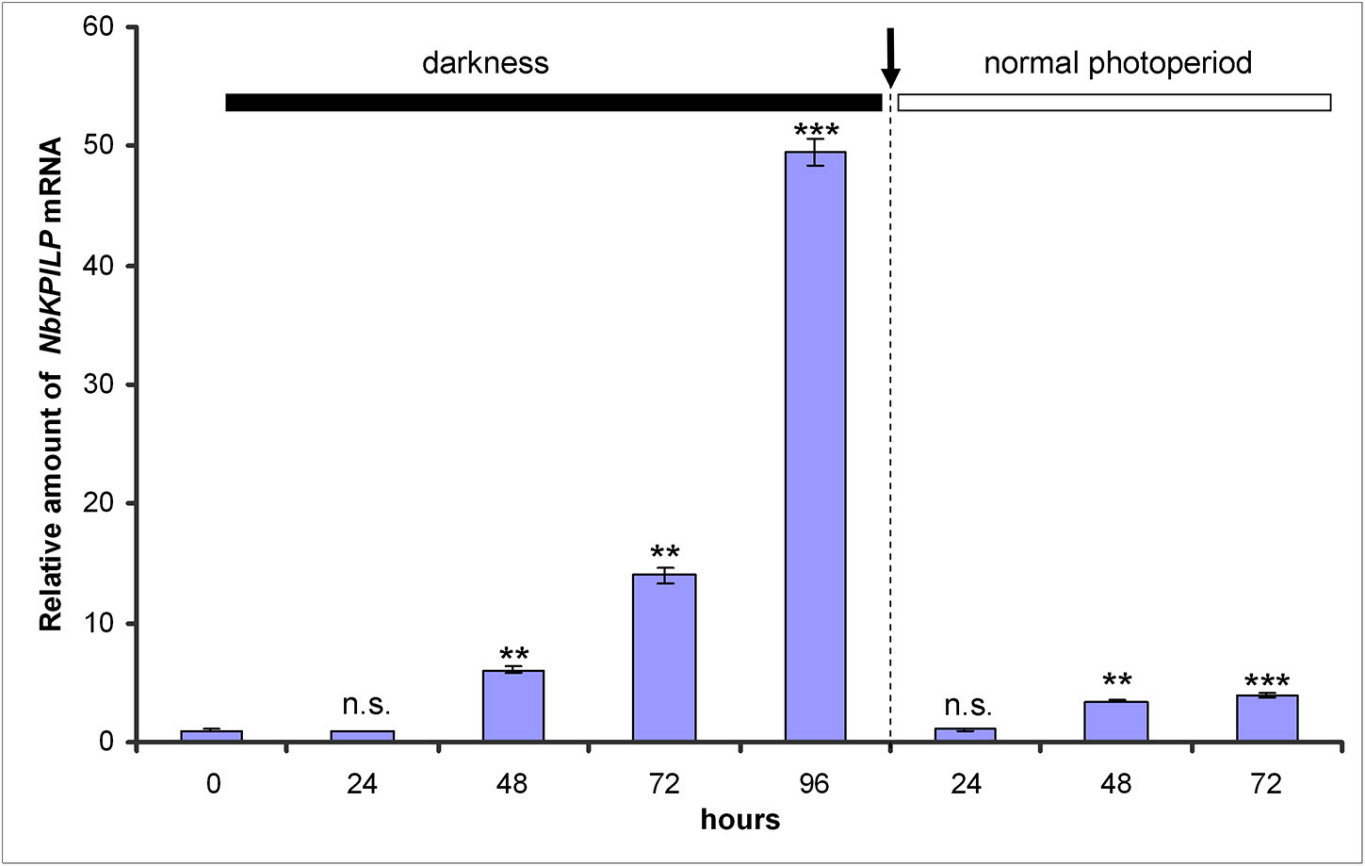
Darkness stimulates the accumulation of *NbKPILP* mRNA in the leaves. Plants were kept in darkness for 24, 48, 72, or 96 h, and the leaf samples were taken for analysis of *NbKPILP* mRNA by qRT-PCR. The data shown represent five independent experiments. The difference in *NbKPILP* mRNA content between control plants and plants after different periods of incubation in darkness (24, 48, 72, 96, and 24 h after return to a normal photoperiod) is shown. ***P < 0.001, n.s. = not significant (Student's *t-*test).

We assumed that like other KPIs (Huang et al., 2010) and KPILP (Suh et al., 2003; Goodwin et al., 2012; Islam et al., 2015a), *NbKPILP* mRNA accumulation is stimulated by the action of abiotic and biotic factors. Therefore, we investigated the effect of abiotic factors such as prolonged darkness on the accumulation of NbKPILP mRNA in leaves. For this, we incubated the plants in darkness for different periods and analyzed *NbKPILP* mRNA content in leaves. Figure 3 shows that after 48 h of darkness *NbKPILP* mRNA content of the leaves significantly increased and continued to increase up to 72 h, reaching almost a 50-fold increase after a 96-h darkness period. However, when the plants were transferred back to a normal light/darkness photoperiod, *NbKPILP* mRNA content returned to the original level. Therefore, prolonged exposure of the plants to darkness induces *NbKPILP* mRNA accumulation in leaves.

**Figure 4:**
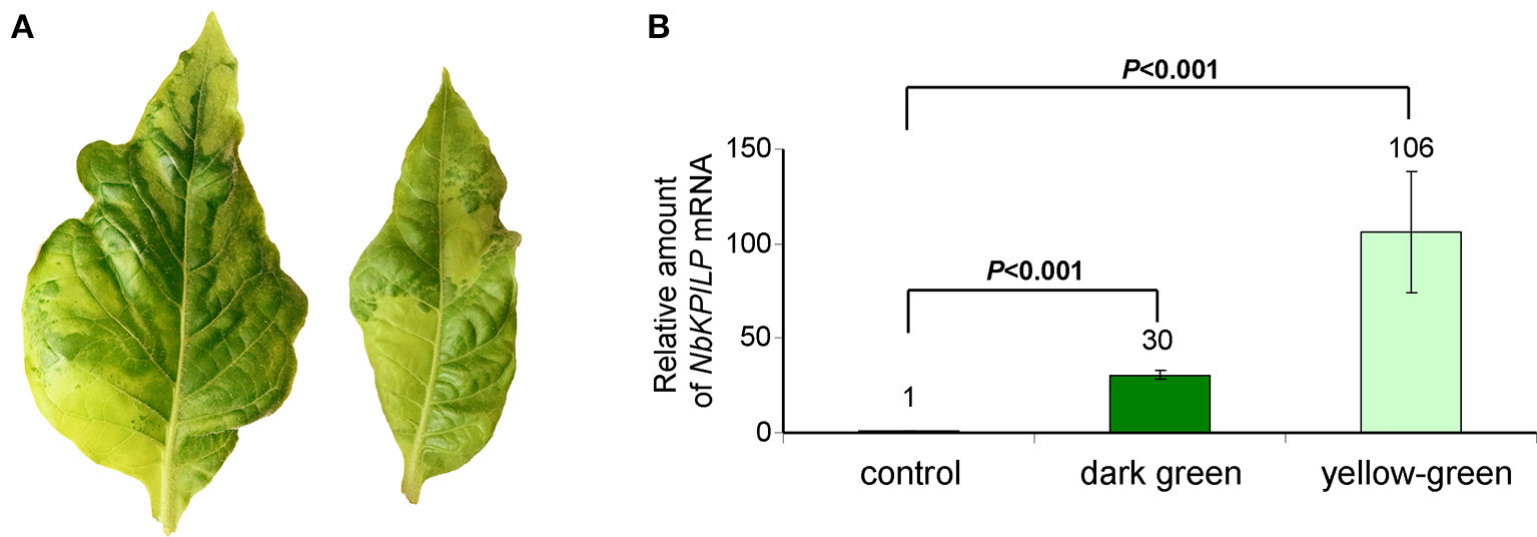
The yellow-green areas of TMV-infected leaves with mosaic symptoms are characterized by a high content of *NbKPILP* mRNA. **(A)** Mosaic symptoms of *N. tabacum* cv. Samsun leaves systemically infected with TMV. **(B)** Relative amounts of *NbKPILP* mRNA in dark-green and yellow-green areas of leaves with mosaic symptoms quantified by qRT-PCR. The data represents five independent experiments, and the standard error bars are indicated. The statistical significance of the difference in *NbKPILP* mRNA content between mosaic areas and control non-infected leaves is shown (Student's *t-*test).

We expected that biotic factors such as TMV infection, which is associated with impaired functioning of the chloroplast and the appearance of mosaic symptoms, should lead to the stimulation of *NbKPILP* mRNA accumulation. To test this hypothesis, we used tobacco (*Nicotiana tabacum* L. cv. Samsun) systemically infected with TMV, whose upper leaves showed mosaic symptoms, i.e., alternation of dark green spots (islands, as described by Atkinson and Matthews, 1970) characterized with a low content of TMV and light, yellow-green leaf areas enriched with TMV particles (Atkinson and Matthews, 1970; Moore et al., 2001; Figure 4A). Our analysis of both regions of mosaic leaves revealed a huge amount of TMV coat protein accumulated in yellow-green islands (data not shown) and a significant increase in *NbKPILP* mRNA content in yellow-green areas compared with dark green islands and especially compared with leaves of the uninfected plants (Figure 4B).

**Figure 5:**
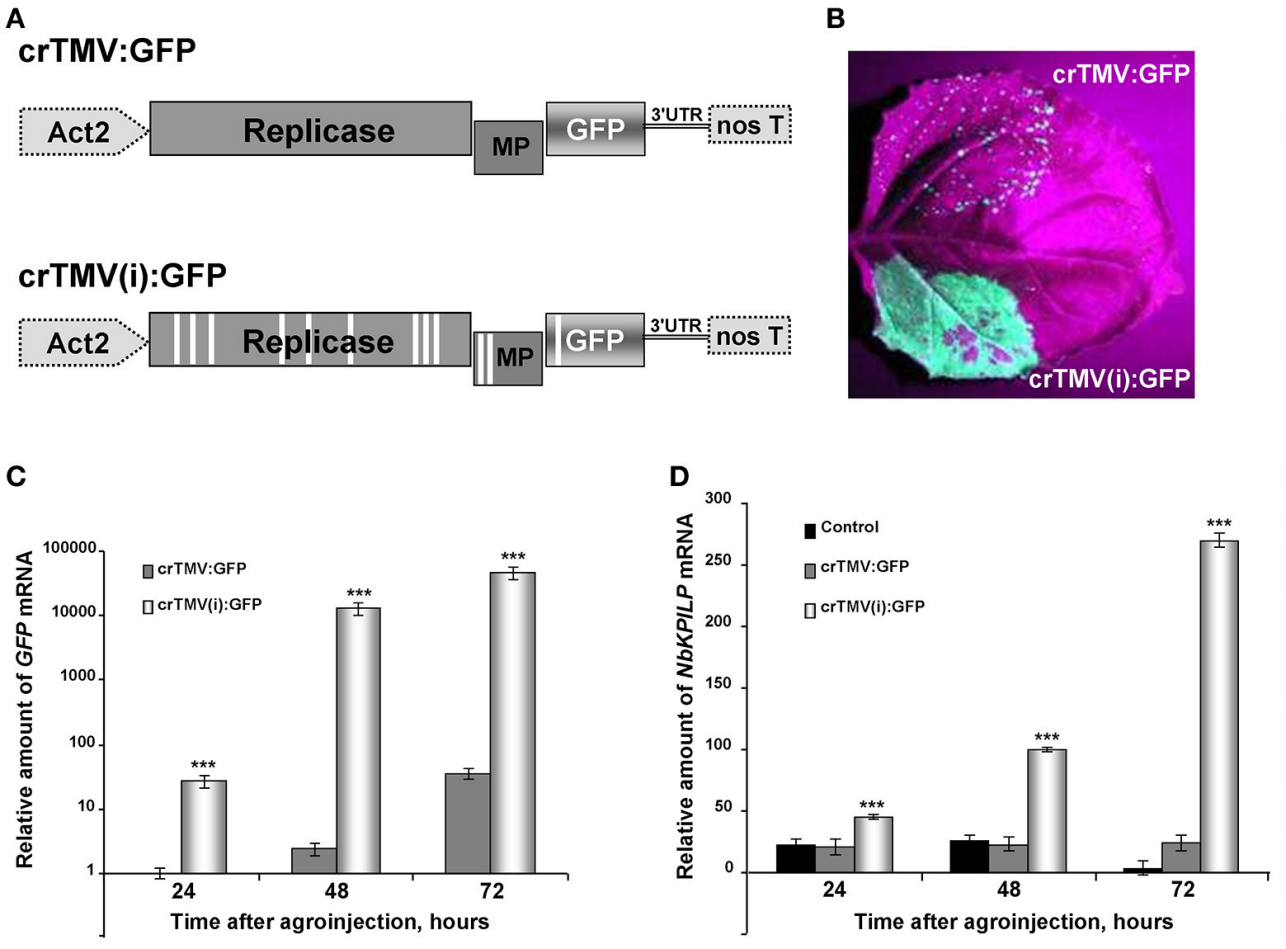
Overproduction of GFP as a foreign protein in the leaves stimulates the accumulation of *NbKPILP* mRNA. **(A)** Schematic representation of the crTMV-based vector crTMV:*GFP* and its intron-containing variant crTMV(i):*GFP*. Act2, *Arabidopsis thaliana act2* gene promotor; genes encoding replicase, movement protein (MP) and GFP, the 3′-untranslated region are shown; nos T stands for terminator of transcription. Introns are denoted by light rectangles. **(B)** UV visualization of GFP in leaves infiltrated with crTMV:*GFP* (upper part of the leaf) and crTMV(i):*GFP* (lower part of the leaf) on the 4th day after infiltration. **(C)** Relative amounts of *GFP* mRNA in *N. benthamiana* leaves agroinjected with crTMV-based vectors quantified by qRT-PCR. *GFP* mRNA accumulation mediated by crTMV:*GFP* vector was set as 1. The statistical significance of the difference between *GFP* mRNA levels mediated by two crTMV-based vectors is shown. ***P < 0.001 (Student's *t-*test). **(D)** Relative amounts of *NbKPILP* mRNA in *N. benthamiana* leaves agroinjected with crTMV-based vectors quantified by qRT-PCR. *NbKPILP* mRNA level of the intact leaf was set as 1. Control, leaf agroinfiltrated with empty vector pBin19. The statistical significance of the difference between *NbKPILP* mRNA levels mediated by two crTMV-based vectors is shown. ***P < 0.001 (Student's *t-*test).

Synthesis of a foreign protein in *N. benthamiana* leaves may also lead to the stimulation of *NbKPILP* mRNA accumulation. We chose GFP as a foreign protein, and used two types of crTMV-based vectors (Figure 5A) with different productive capacities for GFP synthesis in plants. The control vector, crTMV-GFP, provided moderate production of GFP (Figure 5B) and its modified variant, crTMV(i)-GFP, containing multiple introns in the crTMV cDNA sequence (Komarova et al., 2012), mediated excessive GFP production in leaves 4 days after agroinjection (Figure 5B). In parallel with the increase in accumulation of GFP mRNA (Figure 5C), *NbKPILP* mRNA content also increased (Figure 5D).

We concluded that factors of abiotic and biotic stress stimulate *NbKPILP* mRNA accumulation in the leaves.

### *NbKPILP* is a matryoshka gene containing a translatable, alternative nested reading frame encoding a 53-aa polypeptide (53aa-ANRF)

Biologically, the amount of NbKPILP in leaves can be determined by both the transcriptional activity of the gene and the stability of its mRNA in the cytoplasm. Recently, the role of upstream open reading frames (uORFs) in 5′-UTRs in mRNA translation and stability was shown (Tanaka et al., 2016; Bailey-Serres and Ma, 2017; Xu et al., 2017b). To identify putative uORFs, we identified the transcription start site and the sequence of the *NbKPILP* mRNA 5′-UTR using the 5′-Rapid Amplification of Complementary Ends (5′-RACE) approach. As *NbKPILP* mRNA content in the intact leaf is very low (Figure 2), we used two approaches to increase it: (a) incubation of the plants in darkness for 4 days according to the scheme shown in Figure 3 and (b) agroinjection of *N. benthamiana* leaves with a plasmid directing *NbKPILP* mRNA synthesis.

**Figure 6:**
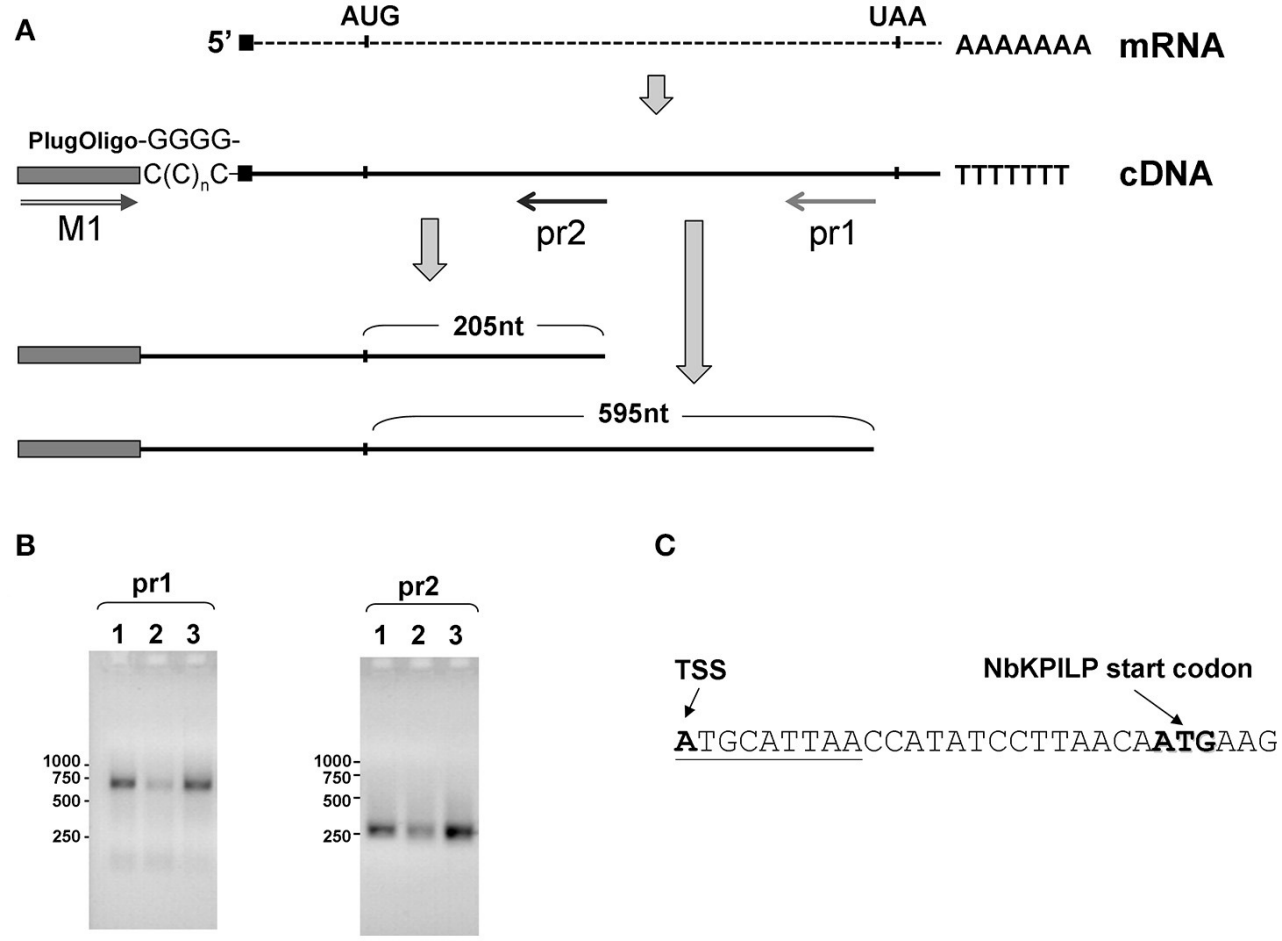
Transcription start site and *NbKPILP* mRNA 5'-UTR identification. **(A)** Schematic representation of the 5′-RACE procedure: cDNA preparation using addition of non-templated dCMP residues to the 3′-end of full-length cDNA by reverse transcriptase (Schmidt and Mueller, 1999) followed by the cDNA second strand synthesis using “PlugOligo” primer and further amplification using “M1” and sequence-specific primers. The position of the primers is marked with arrows. AUG and UAA correspond to start and stop codons of the *NbKPILP* ORF. Sequence complementary to PlugOligo primer designated with gray box; double line with an arrowhead stands for the forward M1 primer; pr1 and pr2, positions of the reverse primers for PCR. **(B)** Agarose gel electrophoresis of PCR products obtained using the adapter primer and pr1 or pr2. Lanes correspond to the following samples: 1—leaves from the plant incubated in darkness for 96 h, 2—leaves from the plant incubated at a normal photoperiod for 24 h after 96-h darkness, 3—leaves 3 days after agroinjection with 35S-*NbKPILP*. **(C)** The nucleotide sequence of the *NbKPILP* mRNA 5′-UTR. The putative uORF is underlined. TSS, transcription start site.

To obtain cDNA suitable for the identification of the 5′-end of *NbKPILP* mRNA, we used the template-switching principle to add an adapter sequence to the 5′-end of the cDNA (Matz et al., 1999). Then, we performed PCR with sequence-specific reverse primers, pr1 or pr2, complementary to the *NbKPILP* region of mRNA, and a forward primer corresponding to the adapter sequence (Figure 6A). We obtained only one PCR product for each reverse primer (pr1 or pr2; Figure 6B). Sequencing of these products allowed us to identify the transcription start site and 5′-UTR (Figure 6C). The mRNA transcription start site is located 23 nts upstream of *NbKPILP* ORF and contains a uORF with the sequence !ATG!CAT!TAA! (Figure 6C). Although it is known that such start-stop codons in the 5′UTR are able to influence the stability of mRNA (Tanaka et al., 2016), we are sure that this is not the case, since the uORF is located directly at the 5′-end. In fact, in agroinjection experiments, comparison of constructs with or without this uORF did not reveal differences in the accumulation of *NbKPILP* mRNA in the leaves (data not shown).

**Figure 7:**
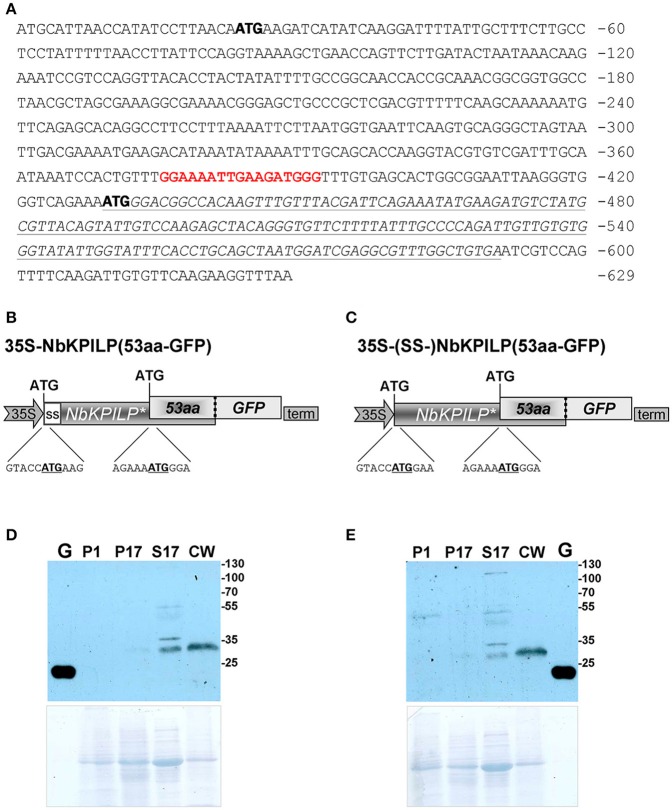
*NbKPILP* mRNA contains an ANRF translated into short 53-aa polypeptide (53aa-ANRF) in *N. benthamiana* leaves. **(A)** The sequence of the *NbKPILP* cDNA with the designated potential start codons (bold). Polypurine block sequence is highlighted in bolded red; *53aa-ANRF* is italicized and underlined. **(B,C)** Schematic representation of the 35S-*NbKPILP(53aa-GFP)* **(B)** and 35S-(SS-)*NbKPILP(53aa-GFP)* **(C)** vectors comprising the following elements: *CaMV* 35S promoter, NbKPILP with or without 24-aa signal sequence (SS-) and 53aa-ANRF fused with GFP. **(D,E)** Western blot analysis using anti-GFP antibodies of *N. benthamiana* leaf proteins at 3 days after agroinjection with the 35S-*NbKPILP(53aa-GFP)* **(D)** or 35S-(SS-)*NbKPILP(53aa-GFP)* **(E)** vectors. Cell homogenate was fractionated into P1, P17, S17, and CW fractions. G, the total soluble protein from leaves 3 days after agroinjection with 35S-GFP vector. The lower panels show the protein loading control stained with Amido Black. All agroinfiltrations were performed in the presence of a vector encoding silencing suppressor P19 from *Tomato bushy stunt virus*.

However, looking closer at the mechanism of *NbKPILP* mRNA translation, we see that the first AUG codon (Figure 7A) is in an unfavorable context and the ribosomal 43S Pre-Initiation Complex (PIC) is likely to skip this codon via the leaky scanning mechanism (Kozak, 1986). For dicots, aaA(A/C)aAUGGCu is the optimal context for the start AUG codon (Joshi et al., 1997; Gupta et al., 2016). The next start AUG codon located 429 nts downstream from the 5′ end of the *NbKPILP* mRNA is in a favorable context (agAaa**AUG**Gga; Figure 7A). The second start AUG codon opens an alternative nested reading frame (ANRF) encoding the 53-aa polypeptide (53aa-ANRF) whose presence can also be predicted for other *KPILP* of *Solanaceae* plants (Figures S3, S4) but which is absent from true KPIs, such as *AtKPI* in *A. thaliana* (Table 1, Table S4).

**Table 1:** *KPILP* matryoshka genes with embedded ANRFs.

**#**	**Plant and protein name**	**Leaf expression**	**EMBL ID**	Identities^*^, %	**Length of the corresponding protein (aa)**	ANRF-encoded peptide contains AH^**^
1	*Nicotiana benthamiana* KPILP (NbKPILP)	No	FN687760	100	201	Yes
2	*Nicotiana glutinosa* KPILP (NgKPILP)	No	AF208022	94.5	229	Yes
3	*Solanum tuberosum* KPILP (StKPILP)	NA	XP_006353918	85.2	198	Yes
4	*Solanum lycopersicum* KPILP (SlKPILP)	NA	XM_004235444	84.8	199	Yes
5	*Trifolium repens* KPI4 (TrKPI4)	No	KF022201.1	28.0	210	Yes
6	*Hevea brasiliensis* KPI *(HbKPI)*	NA	EM_PL:KM979450	36.5	196	Yes
7	*Arabidopsis thaliana* KPI (AtKPI)	NA	At1g73260	33.3	205	No

* *Compared to NbKPILP*.

** *Predicted by http://www.cbs.dtu.dk/services/TMHMM-2.0/*.

According to a prediction by the Phyre2 programme, the 53aa-ANRF contains a transmembrane amphipathic helix (AH) (Table 1, Figure S5) indicating that the hypothetical 53-aa polypeptide is targeted to the cell membrane. To identify whether the 53-aa polypeptide is synthesized, we used an experimental approach in which we fused the 53aa-ANRF encoding sequence with the *GFP* gene, following the general design and context of the *NbKPILP* gene. This technique would allow us to detect 53aa-GFP using a Western blot assay in different cell fractions. We created two vectors encoding the N-terminal part of NbKPILP up to the end of the 53aa-ANRF fused to GFP with a signal sequence [35S-*NbKPILP(53aa-GFP*)] (Figure 7B) and without a signal sequence [35S-*(SS-)NbKPILP(53aa-GFP)*] (Figure 7C). Then, *N. benthamiana* leaves were agroinjected with these constructs and GFP synthesis was examined by fluorescence microscopy which revealed a small amount of fluorescent single cells in leaves (Figure S6). To make sure that the cells synthesized GFP fused with 53-aa polypeptide, we performed a Western blot analysis of subcellular fractions of leaves agroinjected with the abovementioned binary vectors. Figures 7D,E show that in accordance with the predicted amphipathic properties, GFP fused with the 53-aa polypeptide was detected mainly in the cell wall fraction. Its synthesis can be seen in the leaves agroinjected with constructs encoding NbKPILP(53aa-GFP) with (Figure 7D) and without a signal sequence (Figure 7E).

The results of 5′-RACE confirmed that no additional shorter variants of *NbKPILP* cDNA detected in the cell (Figure 6B), therefore the expression of ANRF could not be explained by the existence of a cryptic promoter within the *NbKPILP* gene but is likely to be controlled by the maternal *NbKPILP* mRNA.

We concluded that *NbKPILP* mRNA is capable of directing the synthesis of the 53-aa polypeptide *in vivo*.

### The role of 53aa-ANRF in the accumulation of *NbKPILP* mRNA in *N. benthamiana* leaves

**Figure 8:**
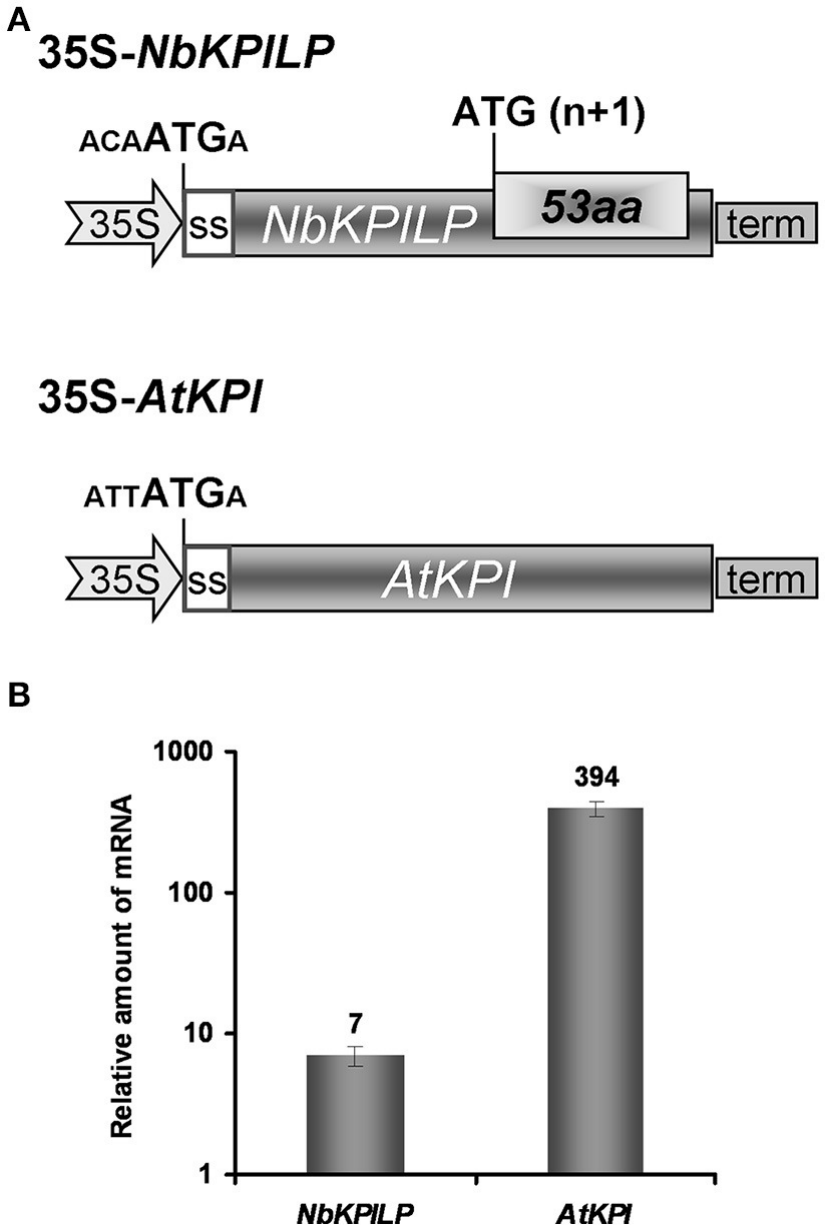
Comparison of *NbKPILP*-encoding mRNA containing ANRF and *AtKPI*-encoding mRNA lacking ANRF accumulation in agroinjected *N. benthamiana* leaves. **(A)** Schematic representation of the 35S-*NbKPILP* and 35S-*AtKPI* constructs. The nucleotide context of the start codon is indicated. SS, signal peptide encoding sequence. **(B)** Relative amount of *NbKPILP* and *AtKPI* mRNA after agroinjection of the corresponding constructs. The level of *NbKPILP* mRNA before agroinjection was set as 1. The mean values with bars indicating SE are shown.

To assess whether ANRF expression affects the level of mRNA accumulation in the leaves, we used two approaches. First, we assumed that the *AtKPI* gene, which does not contain ANRF according to predictions (Table 1), is probably expressed more actively in *N. benthamiana* leaves than *NbKPILP*. We compared the level of mRNA accumulation in agroinjected leaves expressing *NbKPILP* or *AtKPI* from 35S-based constructs (Figure 8A). Figure 8B shows that the level of *AtKPI* mRNA in agroinjected *N. benthamiana* leaves is almost two orders higher than *NbKPILP* mRNA.

**Figure 9:**
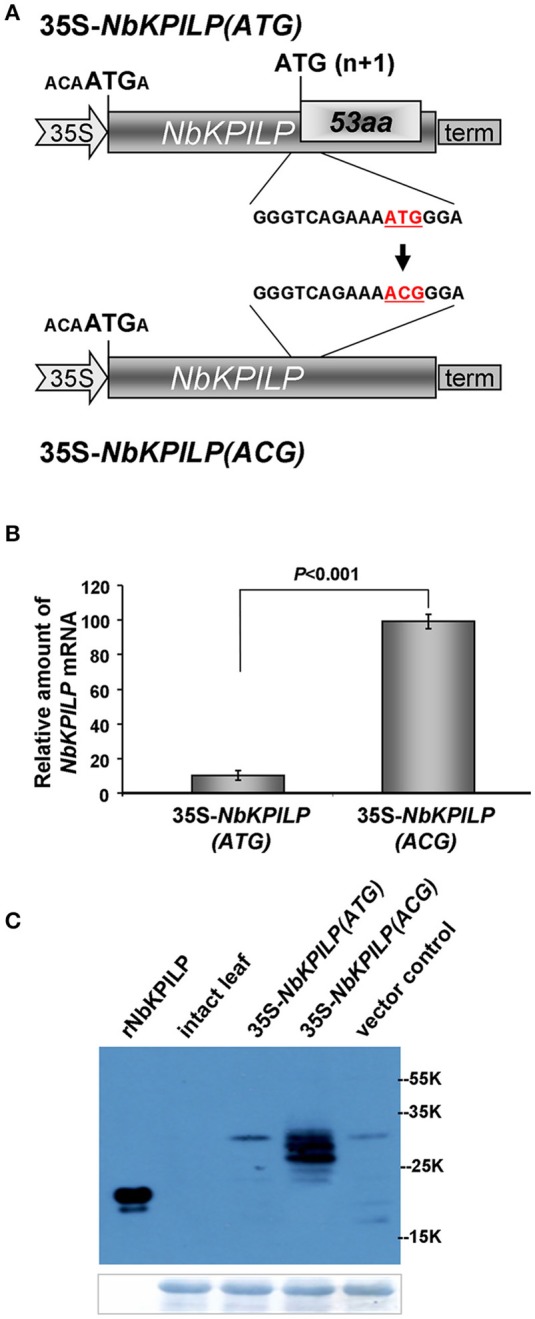
Inactivation of ANRF dramatically increases the accumulation of *NbKPILP* mRNA in the leaves. **(A)** Schematic representation of a 35S-*NbKPILP* vector [35S-*NbKPILP*(ATG)] and 35S-*NbKPILP*(ACG) with the 53aa-ANRF ATG start codon substituted with ACG (both codons are underlined and highlighted in red). **(B)** *NbKPILP* mRNA accumulation in *N. benthamiana* leaves 48 h after agroinjection with 35S-*NbKPILP(ATG)* and 35S-*NbKPILP(ACG)* quantified by qRT-PCR. The values of samples taken just after agroinjection were set as 1. The data represent five independent experiments, and the standard error bars are indicated. The *P*-values were used to assess the statistical significance of differences in *NbKPILP* mRNA levels after agroinjection with 35S-*NbKPILP(ACG)* compared to 35S-*NbKPILP(ATG)*. **(C)** Western blot analysis of total *N. benthamiana* leaf proteins at 48 h after agroinjection with the 35S-*NbKPILP(ATG)* or 35S-*NbKPILP(ACG)* vectors using anti-NbKPILP-6xHis antibodies. rNbKPILP, recombinant NbKPILP with 6xHis-tag produced in *E. coli*. The lower panels show the protein loading control stained with Amido Black.

Then, we hypothesized that the optimal context of the 53aa-ANRF start codon makes it more preferable for scanning PIC, resulting in suppression of recognition and translational initiation from the NbKPILP AUG codon. To test this hypothesis, we inserted a nucleotide substitution into the ANRF AUG start codon, resulting in an ACG codon (Figure 9A) and thus lowering the probability of 53aa-ANRF translation. Figure 9B shows that leaf agroinjection with 35S-NbKPILP(ACG) resulted in a 10-fold increase in mRNA content in comparison to 35S-NbKPILP(ATG). Moreover, the results of a Western blot analysis of leaf proteins were consistent with the mRNA analysis (Figure 9). Antibodies to NbKPILP-6xHis have not revealed NbKPILP-specific proteins in intact leaves. Only after leaf agroinjection with 35S-NbKPILP we detected a weak signal of the 30-kDa protein. For the mutant 35S-NbKPILP(ACG), however, we observed very intense protein bands, including three major and two minor protein bands, the presence of which is likely due to the manifestation of various predicted NbKPILP glycoforms (Figure 1).

We concluded that the accumulation of NbKPILP and its mRNA depends on the expression of 53aa-ANRF.

### Effect of the 53aa-ANRF amphipathic helix (AH) on *NbKPILP* expression

**Figure 10:**
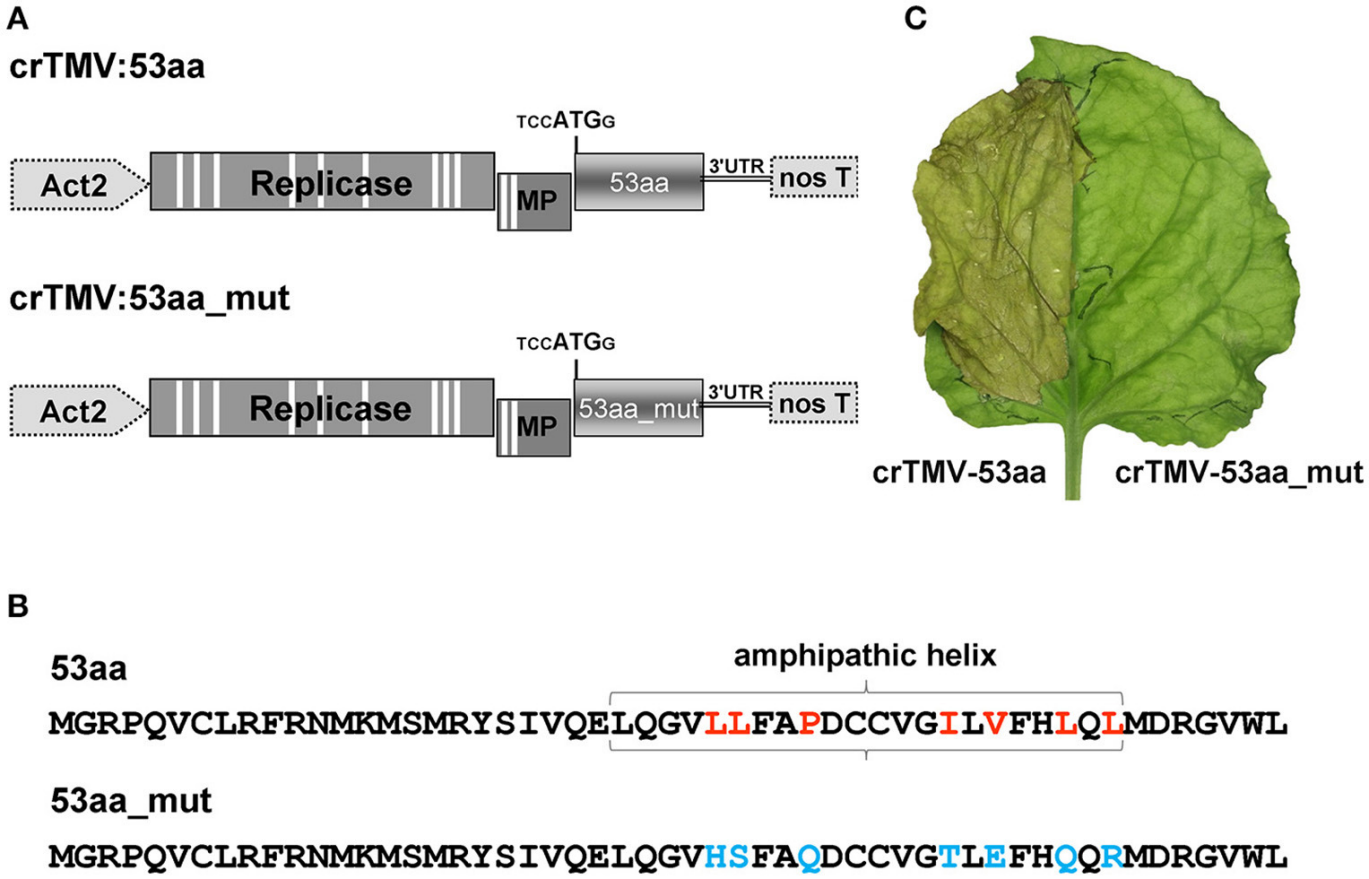
The introduction of a viral vector expressing *53aa-ANRF* into the leaf leads to its necrosis. **(A)** Schematic representation of the crTMV-based vectors expressing ANRF (crTMV-53aa) and its substitution mutant (crTMV-53aa_mut). Act2, *Arabidopsis thaliana act2* gene promoter, genes encoding replicase, movement protein (MP), ANRF (53aa and 53aa_mut) and the 3′-untranslated region are shown; nos T stands for terminator of transcription. Introns are denoted by light rectangles. **(B)** Amino acid sequences of 53aa-ANRF and 53aa_mut-ANRF. Amino acids playing key roles in the formation of the amphipathic helix are marked in red, the substitutions are shown in blue. **(C)** Image of a *N. benthamiana* leaf 4 days after agroinjection with crTMV-53aa (left) and crTMV-53aa_mut (right).

It can be assumed that the inhibitory effect of ANRF on the accumulation of mRNA in the intact leaf is related to the amphipathic nature of the 53-aa polypeptide (Figure S5). It is known that the synthesis of a protein containing AH leads to endoplasmic reticulum membrane modification and to cell death (Hashimoto et al., 2015). To test the potential ability of 53aa-ANRF to cause cellular death, we created two viral vectors based on the crTMV genome (Figure 10A). In the first vector, crTMV-53aa, the coat protein gene was replaced by a sequence encoding 53aa-ANRF. The second vector, crTMV-53aa_mut, encoded a mutant of 53aa-ANRF (53aa_mut-ANRF) containing amino acid substitutions (Figure 10B) that led to the destruction of the AH (Figure S7). Figure 10C shows that the agroinjection of a half-leaf by the crTMV-53aa vector led to necrosis. However, crTMV-53aa_mut, encoding the 53-aa polypeptide without AH, did not induce cell death.

We concluded that 53aa-ANRF is potentially capable of influencing the membrane structure and potentially playing a role in cell death.

**Figure 11:**
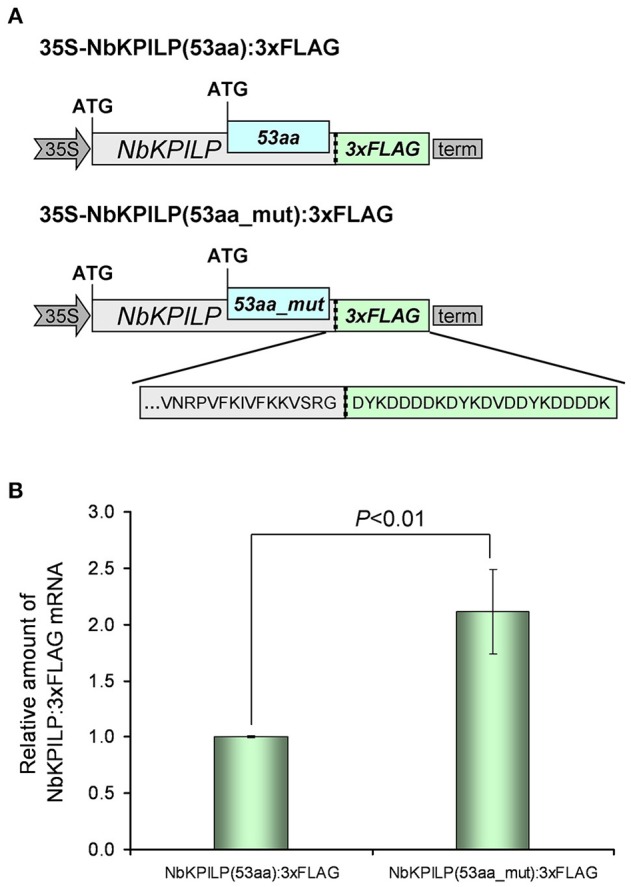
The destruction of the 53aa-ANRF AH leads to an increase in *NbKPILP* mRNA level in the leaf. **(A)** Schematic representation of the 35S-based vectors directing synthesis of NbKPILP fused with the FLAG sequence where 53aa-ANRF contains natural AH [35S-NbKPILP(53aa):3xFLAG] or destroyed AH [35S-NbKPILP(53aa_mut):3xFLAG]. **(B)** Relative amount of NbKPILP:3xFLAG mRNA in *N. benthamiana* leaves agroinjected with 35S-NbKPILP(53aa):3xFLAG or 35S-NbKPILP(53aa_mut):3xFLAG determined by qRT-PCR. The level of NbKPILP(53aa):3xFLAG mRNA was set as 1. Error bars represent SE calculated from three experimental sets of 10–15 leaves each. The *P*-value is used to assess the statistical significance of the differences in the mRNA levels compared with 35S-NbKPILP(53aa):3xFLAG vector taken as a control.

In the next step, we investigated the effect of AH-containing 53aa-ANRF membrane protein synthesis on maternal *NbKPILP* mRNA accumulation in the leaves. To perform agroinjection experiments, we created a 35S-based vector 35S-NbKPILP(53aa):3xFLAG (Figure 11A) which has the natural organization of *NbKPILP* mRNA and allows one to distinguish between the synthesis of endogenous *NbKPILP* mRNA and synthesis of mRNA directed by the 35S-based expression vector. Of note, the injection of *Agrobacterium* leads to the accumulation of endogenous *NbKPILP* mRNA, and, hence, it is necessary to distinguish the experimental mRNA from the endogenous *NbKPILP* mRNA. For this purpose, we added a 3xFLAG-tag encoding sequence (Ueda et al., 2011) to the 3′-terminal end of NbKPILP to obtain an NbKPILP:3xFLAG fusion protein.

To understand the effect of AH on the accumulation of *NbKPILP* mRNA, we created a 35S-NbKPILP(53aa_mut):3xFLAG, that encoded a 53aa_mut-ANRF with amino acid substitutions that cause destruction of AH, but the nucleotide sequence was mutated in a way that preserved the natural amino acid sequence of NbKPILP. To study the relative amount of NbKPILP:3xFLAG mRNA expression, we performed qRT-PCR using FLAG-specific primers (Table S2). Figure 11B shows that removal of the AH from the 53aa-ANRF resulted in more than a two-fold increase in the NbKPILP:3xFLAG mRNA level.

We concluded that the amphipathic nature of the 53-aa polypeptide is responsible for inhibition of *NbKPILP* mRNA accumulation in leaves of intact plants.

## Discussion

Plant KPIs are normally represented by multigene families ranging from several members to several tens of members, as in the case of the poplar (*Populus trichocarpa*), where a wide range of within-family identity was found from low (38%) to high homology (96%) (Major and Constabel, 2008; Philippe et al., 2009). Some members of this family show their activity in organs only under stressful conditions (Rustgi et al., 2017). The upstream regions of the *NbMLP1, NbMLP2*, and *NbMLP3* genes were identified, and promoter elements responsible for defense responses were studied to explain the activation of these genes during stress (Goodwin et al., 2012). Here, our studies of the *N. benthamiana* genome revealed a matryoshka gene organization and another way of regulating gene activity in which ANRF functions as a repressor of a maternal gene in the plant. The *NbKPILP* gene is not unique and its expression pattern indicates no detectable mRNA accumulation in leaves of intact plants, which is also characteristic of other KPIs such as NtKPI1 (Huang et al., 2010), NgKPILP (Suh et al., 2003), TrKPI4 (Islam et al., 2015a), and NbMLP1, NbMLP2, and NbMLP3 (Goodwin et al., 2012). Our analysis revealed that the *NbKPILP* gene belongs to a six-member group, which includes *NgKPILP, StKPILP, SlKPILP, TrKPI4*, and *HbKPI* (Table 1, Table S4). All these genes are characterized by (i) the presence of an ANRF whose start codon is in a more favorable context than the start codon of the main ORF, (ii) the presence of an 18- to 25-nucleotide polypurine block ~30 nts upstream of ANRF, and (iii) the 30- to 53-amino acid long membrane protein encoded by the ANRF.

**Figure 12:**
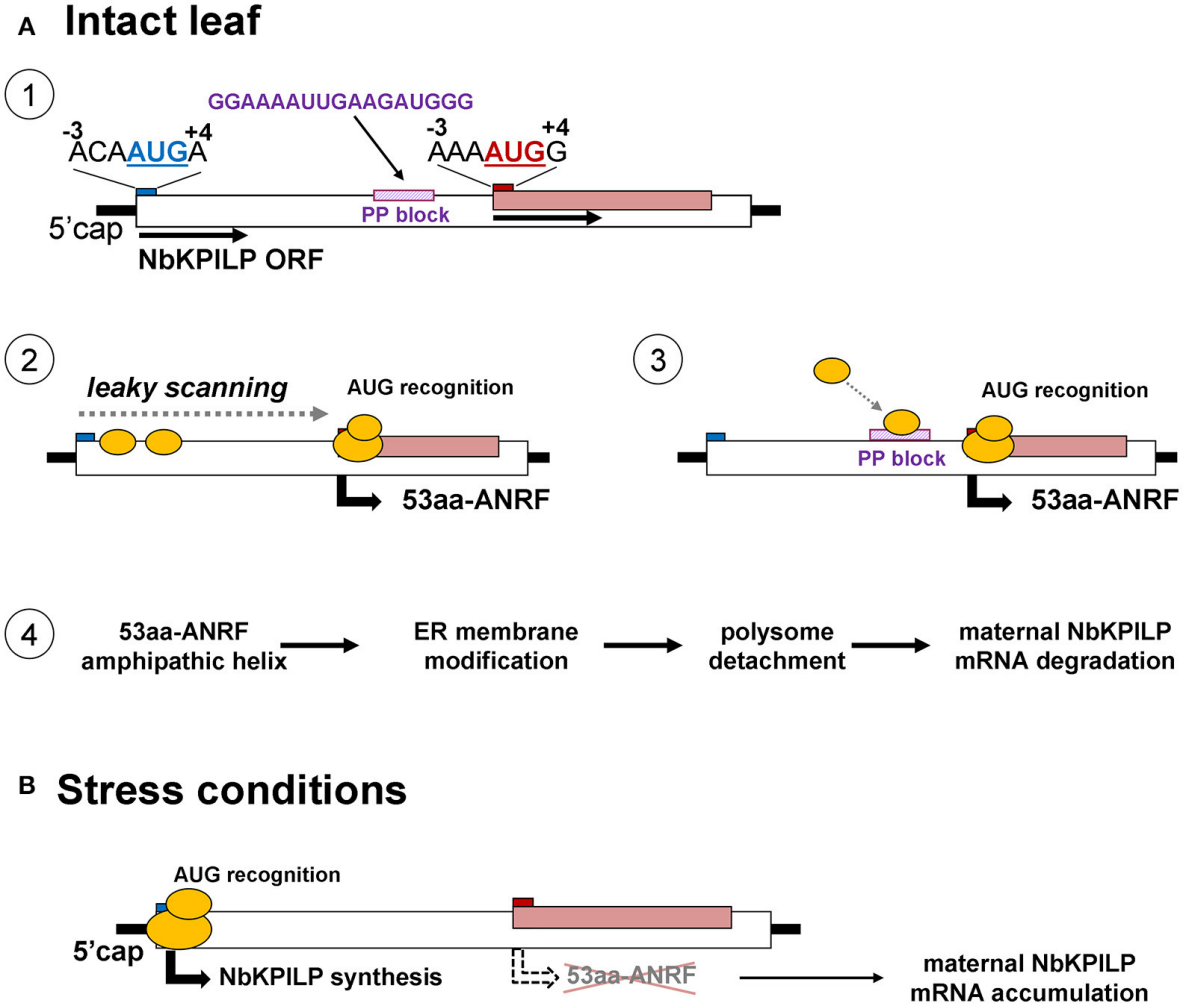
Possible mechanisms for the function of a matryoshka gene in a healthy leaf and after a stressful event. **(A)** Probable mechanism of the 53-aa polypeptide synthesis in the intact leaf. 1. Schematic representation of *NbKPILP* mRNA organization and the context of start codons for the main ORF and ANRF. 2. Initiation of 53aa-ANRF translation via leaky scanning. 3. Initiation of 53aa-ANRF translation via internal ribosomal entry mediated by a polypurine (PP) block. 4. Translation of 53aa-ANRF results in AH-containing 53-aa polypeptide synthesis which leads to polysome detachment from ER and *NbKPILP* mRNA degradation. **(B)** The translation initiation switch from the ANRF start codon to NbKPILP AUG in stress conditions. The initiation of translation is carried out at the first AUG codon resulting in NbKPILP accumulation and downregulation of the 53aa-ANRF synthesis.

In principle, the matryoshka gene design can manifest itself on two levels. In the first level, the chromosome level, the “embedded” gene is located in introns and exons of a larger chromosomal gene. The variety of mechanisms of gene expression is determined not only by the number of protein coding genes but also by the existence of alternative transcription initiation sites and alternative splicing (Mercer and Mattick, 2013; Mercer et al., 2015). Interestingly, unlike in plants, animal genes that encode KPI contain introns (He et al., 2003; Chakrabarty et al., 2006; Isaeva et al., 2012; Chen et al., 2013) and with the help of alternative splicing, can control KPI expression (Sandbrink et al., 1994; Itoh et al., 1999; Kirchhofer et al., 2003; Wood et al., 2014). In plants, there is no alternative splicing for KPIs, so the function of ANRF during mRNA translation can be a mechanism involving stress-regulated gene expression. To explain the low content of NbKPILP and its mRNA in intact leaves, we propose mechanisms that allow the synthesis of the 53-aa polypeptide which modifies ER-membranes in cells of intact leaves (Figure 12). In general, eukaryotic translational initiation is a multistep process that includes PIC assembly, its attachment to the mRNA, mRNA circularization, scanning, start codon selection, and 60S subunit joining (Haimov et al., 2015; Sesma et al., 2017). The first probable mechanism of the 53-aa polypeptide synthesis is based on ribosomal leaky scanning (Figure 12A). Usually, translation is initiated at the first 5′-proximal AUG codon in an acceptable context. The extent of “leakiness” depends on the nucleotide context surrounding AUG. The first AUG codon of *NbKPILP* mRNA is not in the optimal context for dicots (AAN**AUG**GC) (Figure 12A-1; Kim et al., 2014; Gupta et al., 2016), therefore, the formation of the initiating complex is more preferable at the second AUG initiating codon, which is in the favorable context (Figure 12A-2). PIC scanning for over 400 nucleotides is likely to create the risk of ribosome stalling and mRNA degradation (Kawaguchi and Bailey-Serres, 2005; Hsu et al., 2016). Ribosome shunting, which occurs when the ribosome bypasses or shunts parts of the 5′-UTR on its way to the AUG start codon, is unlikely here (Miras et al., 2017; Terenin et al., 2017) since there is a PP block 39 nts upstream of the ANRF AUG codon which can perform the role of an internal ribosome entry site (Dorokhov et al., 2002; Figure 12A-3). In either case, both mechanisms could lead to the synthesis of the 53-aa polypeptide, whose AH is potentially capable of ER-membrane modification, i.e., polyribosome detachment leading to mRNA degradation (Hashimoto et al., 2015; Figure 12A-4). Under stress conditions, the synthesis of the 53-aa polypeptide and the degradation of *NbKPILP* mRNA is downregulated, since the initiation of translation is carried out at the first AUG codon, even in the unfavorable context (Figure 12B). As our experiments showed, stress factors affected the functioning of chloroplasts. It can be assumed that the change in the mode of translation of *NbKPLIP* mRNA under stress is associated with the synthesis or destruction of a certain chloroplast factor, as shown in the study of the uORF participation in the repression of the transcription factor AtHB1 (Ribone et al., 2017). The question arises whether the matryoshka gene organization of the *NbKPILP* gene and the stress-regulating mechanism of its mRNA accumulation in leaves are widespread. Although the current assembly of *N. benthamiana* genome is incomplete, we found ~300,000 sORF in 41,354 transcripts of *N. benthamiana* according to the fifth version of transcriptome (Nakasugi et al., 2014), using the following criteria of a matryoshka gene organization: (a) mRNA contains nested short ORF (translated products between 10 and 100 amino acids), (b) sORF is translatable according to the sORFfinder prediction (Hanada et al., 2010), and (c) AUG codon of the sORF is in favorable Kozak's context (consensus aMN!aug!gS). It turned out that 1,409 ANRFs fall on 1,332 unique transcripts, in which 95% contain single ANRF, 4.4%−2 ANRFs and 0.6%−3 or 4 ANRFs. However, the proportion of ANRF among transcripts with different and identical levels of expression in roots and leaves was practically the same. Thus, the ANRF mechanism is likely to be used mainly for specific genes and tasks.

## Author contributions

YD and TK: conceived of the hypotheses and designed the experiments; ES: performed most of the experiments; TK, AS, and NE: performed some experiments; YD, ES, TK, and AS: evaluated the data and drafted the outline of the manuscript; YD, ES, and TK revised and finalized the manuscript; all the authors read and approved the manuscript.

### Conflict of interest statement

The authors declare that the research was conducted in the absence of any commercial or financial relationships that could be construed as a potential conflict of interest.

## Supplementary material

Figure S1 Molecular phylogenetic analysis of KPIs and KPILPs with highest sequence identity to NbKPILP (see Table S3) calculated from the amino acid sequences by the maximum likelihood method and based on the JTT matrix-based model (Jones et al., 1992). The evolutionary analysis was conducted in MEGA7.

Figure S2 The *NbKPILP* mRNA accumulation in *N. benthamiana*. **(A)** The *NbKPILP* mRNA accumulation in *N. benthamiana* organs according to the *N. benthamiana* Gene Expression Atlas programme (http://sefapps02.qut.edu.au/atlas/tREX6.php). **(B)** Pictures of different ages of *N. benthamiana* plants isolated from soil. **(C)** The *NbKPILP* mRNA accumulation in *N. benthamiana* leaves and roots. The plants of different ages ranging from 2-week-old seedlings to 20-week-old flowering plants shown in **(B)**, were used to analyse *NbKPILP* mRNA by qRT-PCR. The statistical significance of the difference in *NbKPILP* mRNA content between plants of different ages (5, 8, and 20 weeks) and seedlings (2 weeks) is shown. ^**^*P* < 0.01; ^***^*P* < 0.001 (Student's *t*-test).

Figure S3 Nucleotide sequence alignment of *N. benthamiana* Kunitz peptidase inhibitor-like (*NbKPIL*) mRNA (EMBL ID FN687760) with other homologous *Solanaceae* mRNAs: SmKPIL, *Solanum melongena* KPIL (Sol genomics sequence ID Sme2.5_02047.1_g00009.1) NtKPIL (*Nicotiana tabacum* KPIL, Sol genomics sequence ID Ntab-BX_AWOK-SS1956), CaKPIL (*Capsicum annuum* KPIL, sequence ID Ntab-BX_AWOK-SS1956), StKPIL, *Solanum tuberosum* KPIL (EMBL ID XP_006353918), SlKPIL, *Solanum lycopersicum* KPIL (EMBL ID XM_004235444) and NgKPIL, *Nicotiana glutinosa* biotic cell death-associated protein (EMBL ID AF208022). The polypurine block and ANRF are marked by red and yellow, respectively. Asterisks mark the nucleotides which are the same for all sequences. Hyphens represent sequence gaps. The alignment was executed by the Clustal Omega programme (http://www.ebi.ac.uk/Tools/msa/clustalo).

Figure S4 Amino acid sequence alignment of polypeptides encoded by ANRFs.

Figure S5 53aa-ANRF contains an amphipathic helix as predicted by the Phyre2 programme (http://www.sbg.bio.ic.ac.uk/~phyre2/html/page.cgi?id=index).

Figure S6 53aa-GFP localizes predominantly in the CW. GFP imaging by confocal microscopy of *N. benthamiana* leaf at 3 days after agroinjection with the 35S-*NbKPILP(53aa-GFP)* in the presence of a vector encoding silencing suppressor P19 from *Tomato bushy stunt virus*. Images are projections of several confocal sections. Upper panel represents a confocal image superimposed on a bright field image of the same cell.

Figure S7 Amino acid substitutions of 53aa-ANRF **(A)** resulted in AH destruction **(B)** as predicted by the Phyre2 programme.

Table S1 Oligonucleotides used for cloning and 5′-RACE.

Table S2 Oligonucleotides used for qPCR.

Table S3 Kunitz protease inhibitors and Kunitz protease inhibitor-like proteins.

Table S4 *KPILP* matryoshka genes with embedded ANRFs.
