# Supplementary figures and images for: An Alternative Nested Reading Frame May Participate in the Stress-Dependent Expression of a Plant Gene
Source: Front Plant Sci. 2017 Dec 19;8:2137. doi: 10.3389/fpls.2017.02137 (PMC5742262; doi:10.3389/fpls.2017.02137)

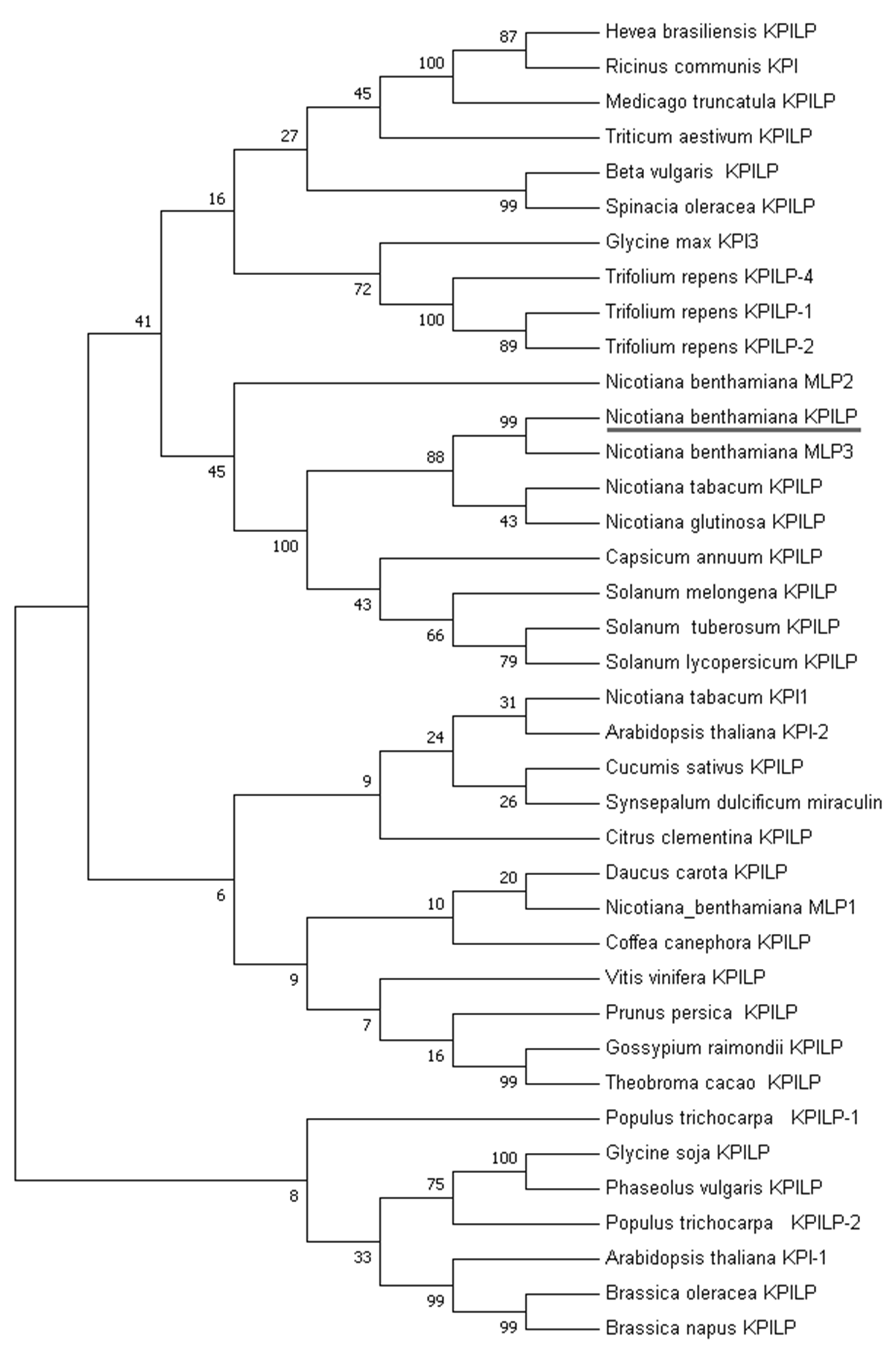

Supplement: Figure S1 — Molecular phylogenetic analysis of KPIs and KPILPs with highest sequence identity to NbKPILP (see Table S3) calculated from the amino acid sequences by the maximum likelihood method and based on the JTT matrix-based model (Jones et al., 1992). The evolutionary analysis was conducted in MEGA7. [file Image1.TIF]

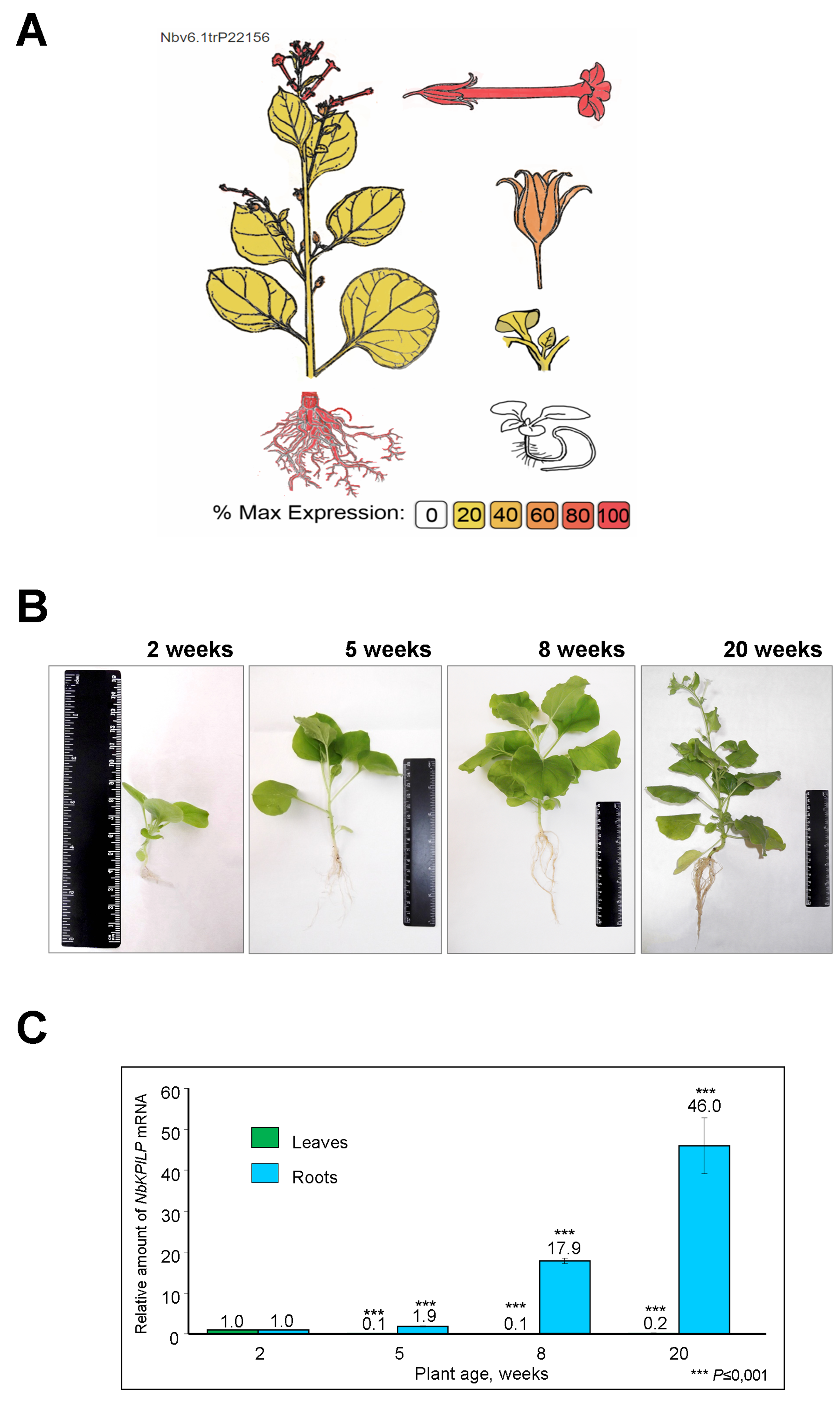

Supplement: Figure S2 — The NbKPILP mRNA accumulation in N. benthamiana. (A) The NbKPILP mRNA accumulation in N. benthamiana organs according to the N. benthamiana Gene Expression Atlas programme (http://sefapps02.qut.edu.au/atlas/tREX6.php). (B) Pictures of different ages of N. benthamiana plants isolated from soil. (C) The NbKPILP mRNA accumulation in N. benthamiana leaves and roots. The plants of different ages ranging from 2-week-old seedlings to 20-week-old flowering plants shown in (B), were used to analyse NbKPILP mRNA by qRT-PCR. The statistical significance of the difference in NbKPILP mRNA content between plants of different ages (5, 8, and 20 weeks) and seedlings (2 weeks) is shown. **P < 0.01; ***P < 0.001 (Student's t-test). [file Image2.TIF]

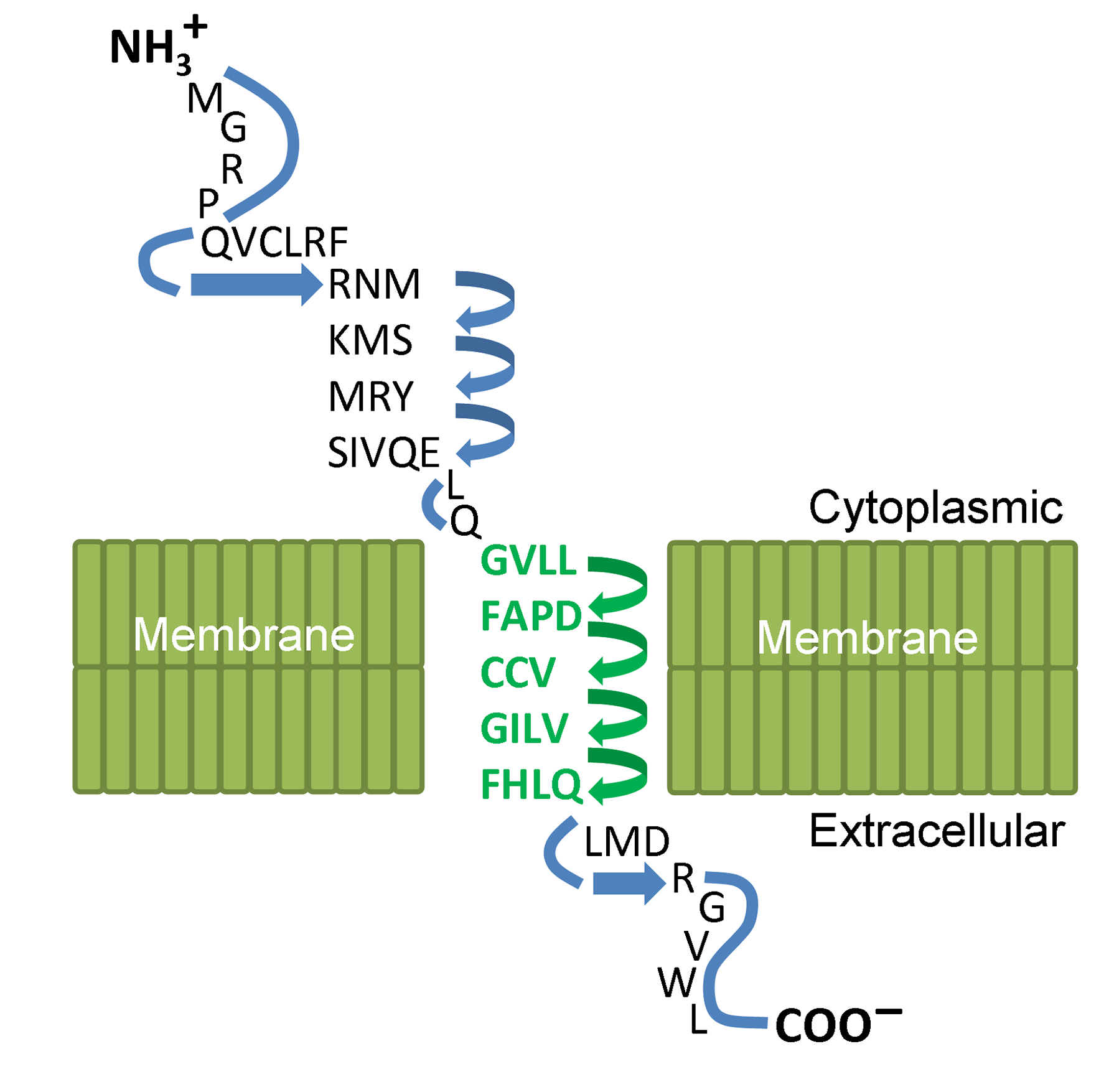

Supplement: Figure S5 — 53aa-ANRF contains an amphipathic helix as predicted by the Phyre2 programme (http://www.sbg.bio.ic.ac.uk/~phyre2/html/page.cgi?id=index). [file Image5.TIF]

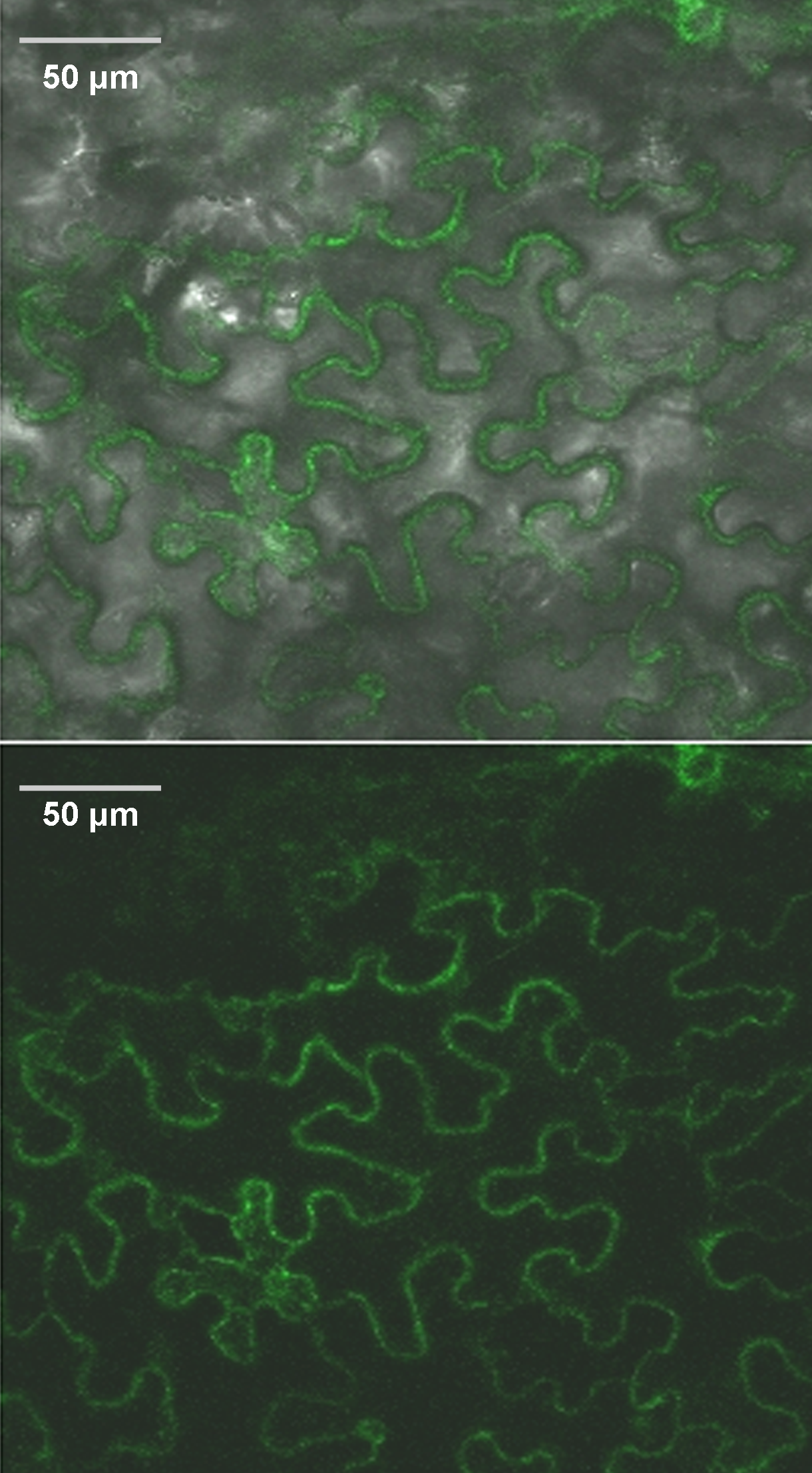

Supplement: Figure S6 — 53aa-GFP localizes predominantly in the CW. GFP imaging by confocal microscopy of N. benthamiana leaf at 3 days after agroinjection with the 35S-NbKPILP(53aa-GFP) in the presence of a vector encoding silencing suppressor P19 from Tomato bushy stunt virus. Images are projections of several confocal sections. Upper panel represents a confocal image superimposed on a bright field image of the same cell. [file Image6.TIF]

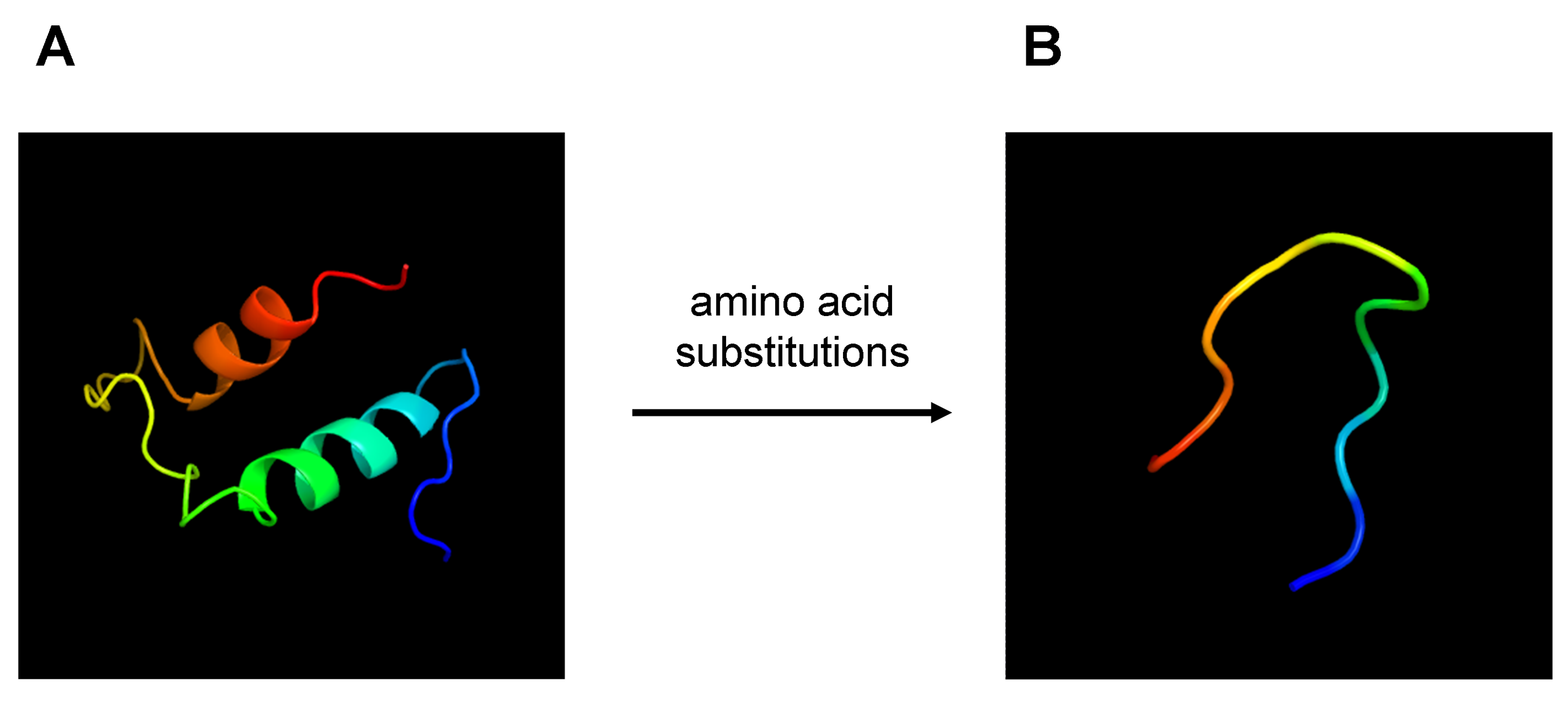

Supplement: Figure S7 — Amino acid substitutions of 53aa-ANRF (A) resulted in AH destruction (B) as predicted by the Phyre2 programme. [file Image7.TIF]
