# Supplementary material for: An Alternative Nested Reading Frame May Participate in the Stress-Dependent Expression of a Plant Gene
Source: Front Plant Sci. 2017 Dec 19;8:2137. doi: 10.3389/fpls.2017.02137 (PMC5742262; doi:10.3389/fpls.2017.02137)
Supplement: Figure S3 — Nucleotide sequence alignment of N. benthamiana Kunitz peptidase inhibitor-like (NbKPIL) mRNA (EMBL ID FN687760) with other homologous Solanaceae mRNAs: SmKPIL, Solanum melongena KPIL (Sol genomics sequence ID Sme2.5_02047.1_g00009.1) NtKPIL (Nicotiana tabacum KPIL, Sol genomics sequence ID Ntab-BX_AWOK-SS1956), CaKPIL (Capsicum annuum KPIL, sequence ID Ntab-BX_AWOK-SS1956), StKPIL, Solanum tuberosum KPIL (EMBL ID XP_006353918), SlKPIL, Solanum lycopersicum KPIL (EMBL ID XM_004235444) and NgKPIL, Nicotiana glutinosa biotic cell death-associated protein (EMBL ID AF208022). The polypurine block and ANRF are marked by red and yellow, respectively. Asterisks mark the nucleotides which are the same for all sequences. Hyphens represent sequence gaps. The alignment was executed by the Clustal Omega programme (http://www.ebi.ac.uk/Tools/msa/clustalo). [file Image3.PDF]

```
SmKPIL      ATGGATGAGTTGGAGAAGAAGAGGGAATTGTTGGTGGAAAGAGATGACTATGCCGG-AAC      59
NgKPIL      ----------------------------------ATGAAGATCATATCAAGGATTTTATT      26
NbKPIL      ----------------------------------ATGAAGATCATATCAAGGATTTTATT      26
NtKPIL      ----------------------------------ATGAAGATCATATCAAGGATTTTATT      26
CaKPIL      -------------------------------------ATGAAGATATCTGGGATTTTATT      23
StKPIL      ----------------------------------------------ATGAAGATTTTATT      14
SlKPIL      ----------------------------------------------ATGAAGATTTTATT      14
                                                               *     *

SmKPIL      AGGTGCTAATAATCATCATGATCCAAAGCCTCCTGGCTCCCAATGGGGAACCGTTGTGGA      119
NgKPIL      GCTTTCTTGCCTTCTATTTTTAACCTTATTC---CAGGTAAAAACTGAACCAGTTCTTGA      83
NbKPIL      GCTTTCTTGCCTCCTATTTTTAACCTTATTC---CAGGTAAAAGCTGAACCAGTTCTTGA      83
NtKPIL      GCTTTCTTGCCTCCTATTTTTAACCTTATTC---CAGGTAAAAGCTGAACCAGTTCTTGA      83
CaKPIL      ACTTTCTTCCCTCCTTAGTTTTACCTTATTCCAAATCACAAAATCCGAACCAGTTGTTGA      83
StKPIL      ACTTTCTTGCTTCCTCTATTTTACCTTATTTCAAATCATAAAATCCGAACCGGTTCTCGA      74
SlKPIL      ACTTTCTTGCTTCCTCTACTTTACCTTATTTCAAACAATAAAATCCGAACCGGTTCTCGA      74
               * **      *         *                 **   * * * *** * **

SmKPIL      TACAAATAATGAACAAGTCCGTCCAGGGATGAGCTACTACATACTCCCCGC---CCCGGG      176
NgKPIL      TACTAATAAACAAGAAATCCGTCCAGGTTACACCTACTACATTTTGCCGGCAACCACCGG      143
NbKPIL      TACTAATAAACAAGAAATCCGTCCAGGTTACACCTACTATATTTTGCCGGCAACCACC--      141
NtKPIL      TACTAATAAACAAGAGGTCCGTCCAGGTTACACCTACTACATTTTGCCGGCAACCACCGC      143
CaKPIL      TACTAACAATGAACAAGTCATGACAGGATCCACCTACTACATATTGCCCGCC---ACCAC      140
StKPIL      TACTAGTAATGAACAAGTCCGTCCGGGGTACACCTACTACATATTGCCCGCT---TCCGC      131
SlKPIL      TACTAATAATGAACAAGTCCGTCCGGGGTACACCTACTACATATTGCCCGCGGCCTCCGC      134
            *** *  **  ** *  **    * **    * ****** **  * ** **     *

SmKPIL      GGGCAACGGGGGTGGCCTAACGCTAGCGAAAGGCGAGAACGGGAGCTGCCCGCTCGACGT      236
NgKPIL      CGCCAACGGCGGTGGCCTAACGCTAGCGAAAGGCGAAAACGGGAGCTGCCCGCTCGACGT      203
NbKPIL      -GCAAACGGCGGTGGCCTAACGCTAGCGAAAGGCGAAAACGGGAGCTGCCCGCTCGACGT      200
NtKPIL      GGCCAACGGCGGTGGCCTAACGCTAGCGAAAGGCGAAAACGGGAGCTGCCCGCTCGACGT      203
CaKPIL      AGGCAACGGTGGTGGCCTAACGCTAGCGCAAGGCGCTAACGGGAGCTGTCCGCTCAACGT      200
StKPIL      GGGCAATGGTGGTGGCCTAACGCTAGCCAAAGGCGAAAACGGGAGCTGCCCCCTCGACGT      191
SlKPIL      GGGCAGTGGTGGTGGCCTAACGCTAGCCAAAGGCGAAAACGGGAGCTGCCCCCTCGATGT      194
             *  *  ** *****************  ******  *********** ** *** * **

SmKPIL      TTTCCAATCACAAAATTCGCAAAGCGTAGGCCTCCCGTTGAAATTCTTGATGGTGAATTC      296
NgKPIL      TTTTCAAGCACAAAATTCACAGAGCAGAGGCCTCCCTTTAAAATTCTTAATGGTGAATTC      263
NbKPIL      TTTTCAAGCAAAAAATGTTCAGAGCACAGGCCTTCCTTTAAAATTCTTAATGGTGAATTC      260
NtKPIL      TTTTCAAGCACAAAATGTTCAGAGCACAGGCCTTCCTTTAAAATTCTTAATGGTGAATTC      263
CaKPIL      TTACCAAGCGCAAAATTCGCAAAGCGTAGGCCTTCCATTGAGATTGTTGATGGTGAATTC      260
StKPIL      TTTTCAAGCACGAAATTCTCAAAGTGTAGGCATCCCGTTGAAATTCTTGATGGTGAATTC      251
SlKPIL      TTTTCAAGCCCGAAATTCTCAAAGTTTAGGCATCCCGTTGAAATTCTTGATGGTGAATTC      254
            **  *** *   ****   ** **   **** * ** ** * *** ** ***********

SmKPIL      CAGCGCTGGCTTACTAGTCGATGAAAATGAAGATATAAATATTAAATTTGCGGCACCAAG      356
NgKPIL      AAGTGCAGGACTAGTAATTGACGAAAATGAAGACATAAATATCAAATTTGCAGCACCAAG      323
NbKPIL      AAGTGCAGGGCTAGTAATTGACGAAAATGAAGACATAAATATAAAATTTGCAGCACCAAG      320
NtKPIL      AAGTGCAGGACTAGTAATTGACGAAAATGAAGACATAAATATAAAATTTGCAGCACCAAG      323
CaKPIL      AAGCTCGGGTTTAGTAATAGACGAAAATGAGGCGATAAATATTAAATTCGCAGCGCCAAA      320
StKPIL      TAGCGCGGGCTTAGTAATCGATGAAAATGAGGACATAAATATAAAATTCGCAGCGCGAAG      311
SlKPIL      TAGCGCGGGTTTAGTAATCGATGAAAATGAAGACATAAATATAAAATTCGCAGCGCAGAG      314
             **  * **  ** ** * ** ******** *  ******** ***** ** ** *  *

SmKPIL      GTTCGTGTCAATATGTAATAGATCAACTGTTTGGAAAATCGAACAAGGAATCGTGACAAC      416
NgKPIL      GTACGTGTCCATTTGCAATAAATCCACTGTTTGGAAAATTGAAGACGGGTTTGTGACCAC      383
NbKPIL      GTACGTGTCGATTTGCAATAAATCCACTGTTTGGAAAATTGAAGATGGGTTTGTGAGCAC      380
NtKPIL      GTACGTGTCAATTTGCAATAAATCCACTGTTTGGAAAATTGAC---GGGTTTGTCACCAC      380
CaKPIL      GTACGAATCAATATGTAAAAAATCAACCGTTTGGAAAATTGAAGAAGGGTTAGTGACAAC      380
StKPIL      GTACGTATCGATTTGTAACGTATCAACTGTTTGGAAAATCGAAGATGGGATTGTGACTAC      371
SlKPIL      GTACGTATCGATTTGTAATGTATCGACTGTTTGGAAAATTGAAGATGGGATTGTGACTAC      374
            ** **  ** ** ** **   *** ** *********** **    **  * ** *  **

SmKPIL      TGGTGGAATTAAAGGTGGATCTGTAAATGGCACGTCTACAAGTTTATTTACGATTCAGAA      476
NgKPIL      TGGCGGAATTAAGGGTGGGTCTGAAAATGGCACGGCCACAAGTTTGTTTACGATTCAGAA      443
NbKPIL      TGGCGGAATTAAGGGTGGGTCAGAAAATGGGACGGCCACAAGTTTGTTTACGATTCAGAA      440
NtKPIL      TGGCGGAATTAAGGGTGGGACTGAAAATGGTACGGCCACAAGTTTGTTTACGATTCAGAA      440
CaKPIL      TGGTGGAATTAAGGGTGGATTAGTAAGTGGTACGTCCACATGTTTATTTACGATTCAGAA      440
StKPIL      TGGTGGAATTAAGGGTGGATCGGAAAATGGCACATCTACAAGTTTATTTACGATTCAGAA      431
SlKPIL      TGGTGGAATTAAGGGTGGATCGGAAAATGGCACGTCTACGAGTTTATTTACGATTCAGAA      434
            *** ******** *****    * ** *** **  * **  **** **************
```

```
SmKPIL          GTATGAAGATGCTTATGCTTTACAATATTGTCCAAGAGCCACAGGATGTTCTTTTATTTG     536
NgKPIL          GTATGAAGATGCCTATGCGTTGCAGTATTGCCCAAGAGCTGCAGGGTGTTCTTTTATTTG     503
NbKPIL          ATATGAAGATGTCTATGCGTTACAGTATTGTCCAAGAGCTACAGGGTGTTCTTTTATTTG     500
NtKPIL          GTATGAAGATGCCTATGCGTTACAGTATTGTCCAAGAGCTACAGGGTGTTCCTTTATTTG     500
CaKPIL          ATATGAAGATGCATATGCTTTACAATATTGGCCTAGACCAAAGGGACGTACTTTTGTTTG     500
StKPIL          ATATGAAGATGCTTATGCTTTACAATATTGTCCTAGAGCTACAGGTTGTTCTTTTATTTG     491
SlKPIL          ATATGAAGATGCTTATGCTTTACAATATTGTCCTAGAGCTACAGGGTGTTCTTTTATTTG     494
                 **********  ***** ** ** ***** ** *** *    **  ** * *** ****

SmKPIL          TCCAAGGTTGTTGTGTGGGTATATTGGTATTTCAACAGCAGCGAATGGAGCCAGGCATTT     596
NgKPIL          CCCCAGATTGTTGTGTGGGTATATTGGTATTGCACCTGCAGCTAATGGGTCGAGGCGTTT     563
NbKPIL          CCCCAGATTGTTGTGTGGGTATATTGGTATTTCACCTGCAGCTAATGGATCGAGGCGTTT     560
NtKPIL          TCCAAGATTGTTGTGTGGGTATATTGGTATTGCACCTGCAGCTAATGGATCGAGGCGTTT     560
CaKPIL          TCCAAAATTGTCCTGTGGGTATATTGGTATTTCACCAGTTGCAAATGGATCGAGGCGTTT     560
StKPIL          TCCAAGATTGTTGTGTGGGTATATTGGTATTTTACCAGCTGAAAATGGATCGAGGCATTT     551
SlKPIL          TCCAAGATTGCTGTGTGGGTATATTGGTATTTTAACAGCGGAAAATGGATCGAGGCATTT     554
                 ** *  ***   ******************  * * *  *  *****  * **** ***

SmKPIL          GGCGGTGAACCGGCCGGTTTTTAAGATTGTGTTCAGGAAGGCCTAA--------------     642
NgKPIL          GGCTGTAAATCGTCCAATTTTCAAGATTGAGTTCAAGAAGGCCTCAGATACAGAAGTGAA     623
NbKPIL          GGCTGTGAATCGTCCAGTTTTCAAGATTGTGTTCAAGAAGGTTTAA--------------     606
NtKPIL          GGCTGTGAATCGTCCAGTTTTCAAGATTGTGTTCAAAAAGGCTTAA--------------     606
CaKPIL          GGCTGTGAACCGAACGGCTTGCAAGATTGTGTTCAAGAAAGCCTAA--------------     606
StKPIL          GGCTGTGAACCGGCCGGTTTTCAAGATTGTGTTCAGGAAGGCATAA--------------     597
SlKPIL          GGCTGTGAACCGGCCGGTTTTCAAGATTGTGTTCAGGAAGGCATAA--------------     600
                *** ** ** **   *   **  ******* *****  ** *  * *

SmKPIL          ------------------------------------------------------------     642
NgKPIL          TTTTTCACAATTTGATATTTATAATAGATGTGGATTTTCTAATACTTATATGAACAAAAG     683
NbKPIL          ------------------------------------------------------------     606
NtKPIL          ------------------------------------------------------------     606
CaKPIL          ------------------------------------------------------------     606
StKPIL          ------------------------------------------------------------     597
SlKPIL          ------------------------------------------------------------     600


SmKPIL          -------642
NgKPIL          TTATTAG690
NbKPIL          -------606
NtKPIL          -------606
CaKPIL          -------606
StKPIL          -------597
SlKPIL          -------600
```
