# Supplementary material for: An Alternative Nested Reading Frame May Participate in the Stress-Dependent Expression of a Plant Gene
Source: Front Plant Sci. 2017 Dec 19;8:2137. doi: 10.3389/fpls.2017.02137 (PMC5742262; doi:10.3389/fpls.2017.02137)
Supplement: Figure S4 — Amino acid sequence alignment of polypeptides encoded by ANRFs. [file Image4.PDF]

NtKPILP	------------MKMHMLYNIGLDQRDVLLFVQNCPVGILVFHQLQMDRGVWL	41
CaKPILP	------------MKMHMLYNIVQEQKDVLLFVQNCRVDILVFYQLLMDRSVWL	41
NbKPILP	MGRPQVCLRFRNMKMSMRYSIVQELQGVLLFAPDCCVGILVFHLQLMDRGVWL	53
NgKPILP	MARPQVCLRFRSMKMPMRCSIAQELQGVLLFAPDCCVGILVLHLQLMGRGVWL	53
SmKPILP	MARLQVYLRFRSMKMLMLYNIVQEPQDVLLFVQGCCVGILVFQQQRMEPGIWR	53
StKPILP	MAHLQVYLRFRNMKMLMLYNIVLELQVVLLFVQDCCVGILVFYQLKMDRGIWL	53
SlKPILP	MARLRVYLRFRNMKMLMLYNIVLELQGVLLFVQDCCVGILVF-----------	42
