# Supplementary material for: An Alternative Nested Reading Frame May Participate in the Stress-Dependent Expression of a Plant Gene
Source: Front Plant Sci. 2017 Dec 19;8:2137. doi: 10.3389/fpls.2017.02137 (PMC5742262; doi:10.3389/fpls.2017.02137)
Supplement: Table S1 — Oligonucleotides used for cloning and 5′-RACE. [file Table1.DOC]

| Primer | Nucleotide sequence |
| --- | --- |
| ss-NbKPILP(SacI)d | GAGCTCGAACCAGTTCTTGATACTAA |
| NbKPILP(HindIII)r | AAGCTTTTAAACCTTCTTGAACACAAT |
| AtKPI(BamHI)d | CTGGCCTCAAACGCATATGGT |
| AtKPI(HindIII)r | CTGCAGTCACATAGTCTTGGA |
| NbKPILP(KpnI)d | GGTACCATGAAGATCATATCAAGGA |
| NbKPILP(SalI)r | GTCGACTTAAACCTTCTTGAACACAAT |
| AtKPI(SacI)d | GAGCTCATGACAAAAACTACCAAAAC |
| AtKPI(PstI)r | TTTCGTCCAAGACTATGTGACTGCAG |
| 53aa_end(BamHI)r | GGATCCCAGCCAAACGCCTCGATCCAT |
| 53aa(ACG)d | TCAGAAAACGGGACGGCCACAAGTT |
| 53aa(ACG)r | GGCCGTCCCGTTTTCTGACCCAC |
| 53aa(NcoI)d | CCATGGGACGGCCACAAGTTTG |
| 53aa(XhoI)r | CTCGAGTCACAGCCAAACGCCTCG |
| 53aa(AHmut)d | GTTGTGTGGGTACATTGGAATTTCACCAGCAGCGAATGG |
| 53aa(AHmut)r | CCCACACAACAATCTTGGGCAAATGAATGAACACC |
| 3xFlag(XbaI)d | TCTAGAGGAGACTATAAAGACGACGATGATAAAGACTACAAGGATGACGATAAG |
| 3xFlag(SalI)r | GTCGACTATTTATCGTCGTCGTCCTTATAATCCTTATCGTCATCCTTGTAGTC |
| NbKPILP(XbaI)r | TCTAGAAACCTTCTTGAACACAATC |
| pr1 | TCACAGCCAAACGCCTCGATC |
| pr2 | CCGTTTTCGCCTTTCGCTAGC |
| PlugOligo | AAGCAGTGGTATCAACGCAGAGTACGGGGG |
| M1 | AAGCAGTGGTATCAACGCAGAGT |

**Table S1. Oligonucleotides used for cloning and 5’-RACE**
