# Supplementary material for: An Alternative Nested Reading Frame May Participate in the Stress-Dependent Expression of a Plant Gene
Source: Front Plant Sci. 2017 Dec 19;8:2137. doi: 10.3389/fpls.2017.02137 (PMC5742262; doi:10.3389/fpls.2017.02137)
Supplement: Table S2 — Oligonucleotides used for qPCR. [file Table2.DOC]

**Table S2. Oligonucleotides used for qPCR**

| **Gene** | **Forward Primer** | **Reverse Primer** | **PCR Size, bp** | **Acquisition temperature,^o^C** |
| --- | --- | --- | --- | --- |
| 18S rRNA | ACGGCTACCACATCCAAG | ACTCATTCCAATTACCAGACTC | 116 | 50 |
| NbKPILP | TGAGCACTGGCGGAATTAAGG | ATACCAATATACCCACACAACAATCTG | 157 | 53 |
| GFP | GCAGAAGAACGGCATCAAG | GCTCAGGTAGTGGTTGTCG | 138 | 52 |
| NbKPILP:3xFLAG | GATTGTTGTGTGGGTATATTGG | CATCGTCGTCTTTATAGTCTCC | 126 | 51 |
| AtKPI | GAATCACAGAACCTCAACATC | AACGAATCTTGTCCGAACC | 149 | 54 |
