# Supplementary material for: An Alternative Nested Reading Frame May Participate in the Stress-Dependent Expression of a Plant Gene
Source: Front Plant Sci. 2017 Dec 19;8:2137. doi: 10.3389/fpls.2017.02137 (PMC5742262; doi:10.3389/fpls.2017.02137)
Supplement: Table S3 — Kunitz protease inhibitors and Kunitz protease inhibitor-like proteins. [file Table3.DOC]

Table S3. Kunitz protease inhibitors and Kunitz protease inhibitor-like proteins

| # | Plant and protein name | Plant family | UniProtKB ID | Identities, % | Length  (aa) | Sequence (aa) |
| --- | --- | --- | --- | --- | --- | --- |
|  |  |  |  |  |  |  |
| 1 | *Nicotiana benthamiana* KPILP  (NbKPILP) | *Solanaceae* | D4IHB9 | 100 | 201 | >NbKPILP  MKIISRILLLSCLLFLTLFQVKAEPVLDTNKQEIRPGYTYYILPATTANGGGLTLAKGENGSCPLDVFQAKNVQSTGLPLKFLMVNSSAGLVIDENEDINIKFAAPRYVSICNKSTVWKIEDGFVSTGGIKGGSENGTATSLFTIQKYEDVYALQYCPRATGCSFICPRLLCGYIGISPAANGSRRLAVNRPVFKIVFKKV  >NbKPILP ANRF  MGRPQVCLRFRNMKMSMRYSIVQELQGVLLFAPDCCVGILVFHLQLMDRGVWL |
| 2 | NbMLP3=NbKPILP |  | TC17637*** | 100.0 | 201 | >NbMLP3  MKIISRILLLSCLLFLTLFQVKAEPVLDTNKQEIRPGYTYYILPATTANGGGLTLAKGENGSCPLDVFQAKNVQSTGLPLKFLMVNSSAGLVIDENEDINIKFAAPRYVSICNKSTVWKIEDGFVSTGGIKGGSENGTATSLFTIQKYEDVYALQYCPRATGCSFICPRLLCGYIGISPAANGSRRLAVNRPVFKIVFKKV |
| 3 | *Nicotiana tabacum* KPILP |  | Ntab-BX_AWOK-SS1956* | 96.5 | 201 | >NtKPILP  MKIISRILLLSCLLFLTLFQVKAEPVLDTNKQEVRPGYTYYILPATTAANGGGLTLAKGENGSCPLDVFQAQNVQSTGLPLKFLMVNSSAGLVIDENEDINIKFAAPRYVSICNKSTVWKIDGFVTTGGIKGGTENGTATSLFTIQKYEDAYALQYCPRATGCSFICPRLLCGYIGIAPAANGSRRLAVNRPVFKIVFKKA |
| 4 | *Nicotiana glutinosa* KPILP |  | Q850R9 | 94.5 | 229 | >NgKPILP  MKIISRILLLSCLLFLTLFQVKTEPVLDTNKQEIRPGYTYYILPATTGANGGGLTLAKGENGSCPLDVFQAQNSQSRGLPLKFLMVNSSAGLVIDENEDINIKFAAPRYVSICNKSTVWKIEDGFVTTGGIKGGSENGTATSLFTIQKYEDAYALQYCPRAAGCSFICPRLLCGYIGIAPAANGSRRLAVNRPIFKIEFKKASDTEVNFSQFDIYNRCGFSNTYMNKSY  >NgKPILP ANRF  MARPQVCLRFRSMKMPMRCSIAQELQGVLLFAPDCCVGILVLHLQLMGRGVWL |
| 5 | *Solanum tuberosum* KPILP |  | M1AMX7 | 85.2 | 198 | >StKPILP  MKILLLSCFLYFTLFQIIKSEPVLDTSNEQVRPGYTYYILPASAGNGGGLTLAKGENGSCPLDVFQARNSQSVGIPLKFLMVNSSAGLVIDENEDINIKFAARRYVSICNVSTVWKIEDGIVTTGGIKGGSENGTSTSLFTIQKYEDAYALQYCPRATGCSFICPRLLCGYIGILPAENGSRHLAVNRPVFKIVFRKA  >StKPILP ANRF  MAHLQVYLRFRNMKMLMLYNIVLELQVVLLFVQDCCVGILVFYQLKMDRGIWL |
| 6 | *Solanum lycopersicum* KPILP |  | K4BJT7 | 84.8 | 199 | >SlKPILP  MKILLLSCFLYFTLFQTIKSEPVLDTNNEQVRPGYTYYILPAASAGSGGGLTLAKGENGSCPLDVFQARNSQSLGIPLKFLMVNSSAGLVIDENEDINIKFAAQRYVSICNVSTVWKIEDGIVTTGGIKGGSENGTSTSLFTIQKYEDAYALQYCPRATGCSFICPRLLCGYIGILTAENGSRHLAVNRPVFKIVFRKA  >SlKPILP ANRF  MARLRVYLRFRNMKMLMLYNIVLELQGVLLFVQDCCVGILVF |
| 7 | *Solanum melongena* KPILP |  | Sme2.5_02047.1_g00009.1* | 75.12 | 213 | >SmKPILP  MDELEKKRELLVERDDYAGTGANNHHDPKPPGSQWGTVVDTNNEQVRPGMSYYILPAPGGNGGGLTLAKGENGSCPLDVFQSQNSQSVGLPLKFLMVNSSAGLLVDENEDINIKFAAPRFVSICNRSTVWKIEQGIVTTGGIKGGSVNGTSTSLFTIQKYEDAYALQYCPRATGCSFICPRLLCGYIGISTAANGARHLAVNRPVFKIVFRKA |
| 8 | *Capsicum annuum*  KPILP |  | CA03g23560* | 73.5 | 201 | >CaKPILP  MKISGILLLSSLLSFTLFQITKSEPVVDTNNEQVMTGSTYYILPATTGNGGGLTLAQGANGSCPLNVYQAQNSQSVGLPLRLLMVNSSSGLVIDENEAINIKFAAPKYESICKKSTVWKIEEGLVTTGGIKGGLVSGTSTCLFTIQKYEDAYALQYWPRPKGRTFVCPKLSCGYIGISPVANGSRRLAVNRTACKIVFKKA |
| 9 | *Nicotiana benthamina* miraculin-like protein (NbMLP1) |  | F2VJF4 | 39.47 | 205 | >NbMLP1  MKTKQLFLPFLIFTXSFNSFLSSAAEAPPAVVDIAGKKLRTGIDYYILPVVRGRGGGLTLDSTGNESCPLDAVVQEQKEINNGFPLTFTPVNPKKGVIRESTDLNIKFSAASICVQTTLWKLDDFDETTGKYFITIGGNEGNPGRETISNWFKIEKFERDYKLVYCPTVCNFCKVICKDIGIFIQDGTRRLALSDVPFKVMFKKA |
| 10 | NbMLP2 |  | TC17973*** (NbMLP2) | 38 |  | >NbMLP2  MVPFLLVALSTSSFFLVKAQDVPEPVLDVSGNPVRTGVNYFILPAGRGNGGGLQVASIRNRTNPLVVSQHADESSIGGYLQFSPVNPNENIIRISTDLNVKFTSIHISDSSTVWRINTEIIPQRYLVTVGGVEGNPGRETLSNWFSIDRYEDAYKLVYCPGVCETCRPFCGDIGILVEGSKRVLFLRFDKPLKVTFHKI |
| 11 | *Nicotiana tabacum* KPI1  (NtKPI1) |  | B8Y888 | 33.2 | 209 | >NtKPI1  MNTLLLLLSLSVIPIALCVPNPSRFLAGSSPSPVLDINGDKVKVGLNYFVLPVIRGRGGGLLPSNVKQNNTCPRDIIQNSDEVQEGLPVVFAPFNTKKGVVRLSIDLNVRFFTPTICARETIWKLGTYDDKLKQYFIVTGGVEGNPGPQTLSSWFKIEKLGTDYKFVFCPSVCKICKVICKDVGIYTKDGVRFLALSDTPLRVMFKKTF |
| 12 | Vitis vinifera KPILP | *Vitaceae* | A5AXN3 | 47.6 | 203 | >VvKPI  MKTTSLLFSLLLIALAVKPFSVAAESAPDPVLDTEGKKLRSGVDYYILPVFRGRGGGLTLASTGNESCPLDVVQEQQEVSNGLPLTFTPVNPKKGVIRVSTDHNIKFSASTICVQSTLWKLEYDESSGQWFVTTGGVEGNPGRETLDNWFKIEKYEDDYKLVFCPTVCDFCKPVCGDIGIYIQNGYRRLALSDVPFKVMFKKA |
| 13 | *Prunus persica* KPILP | Rosaceae | M5X024 | 42.6 | 208 | >PpKPILP  MALKAPYPLVFCFLLFAFSAKLRSVAADAAPSPVLDITGNKLQTGVDYYILPVIRGRGGGLTLASTSNKTSCPLDVVQEQNEVSNGLPLKFSPVNVTKGVVRVSTDLNIKFSATTICVQSTVWKLGKFDEQTGQWFVTSGGVEGNPGRQTTSNWFKIEKFGDDYKLVFCPTVCNFCKVICGDVGIFFQDGKRRLALSDVPFRAMFKKV |
| 14 | Populus trichocarpa  KPILP-1 | Salicaceae | U5FKJ0 | 42.1 | 206 | >PtKPI-1  MRAAILALSFLLFALAANQLPRVAATAAPEPVLDVTGKILRTGTSYYILPVIRGRGGGLKMASTVRRTCPLDVVQDRYEASNGLPLKFTPVNTKKGVVRVHTDLNIRFSAGSICHQSTAWKLDNYDEWTKQWFVTTDGVEGNPGPETTNNWFKIEKFEDKYKLVFCPTVCQHCKVMCKDIGIYVDAKGVRRLALTNVPLKVMFKKA |
| 15 | Populus trichocarpa  KPILP-2 |  | U5FH02 | 32.4 | 209 | >PtKPI-2  MNYPMLLLCLLLLAFACTKQSIAAAEPVLDIDGEKLVAGTEYYILPVFRGRGGGITMASNKTSCPLAVVQDRLEVSKGLPLTFTPAADDKKGVILVSTDLNIKFLAKTTCPQSTVWKITKSSNSKVQWFVSTGGVEGNPGFNTVTNWFQIEKADDDYKIVFCPTKVCNCGVLCRDIGIYIEDNGTRTLSLSDALQPFKVQFKKALKKNS |
| 16 | *Gossypium Raimondii* KPILP | *Malvaceae* | A0A0D2THF4 | 41.7 | 198 | >GRKPI  MKTALFLAITSLVLGSTIASDEFDPVLDISGQELRTGIDYYILPVIRGRGGGLTLASTGNETCPLDVVQEQQEVSNGLPLTFSPVNVTQGVVRVSTDLNIKFSAASICVQTTLWTLRFDESVQKYVVTTGGVEGNPGRETLSNWFKIQKFEDDYRLVYCPTVCNFCRPVCGALGVFMDGGTRRLAISDEPLKVMFKRA |
| 17 | *Theobroma cacao* KPILP |  | A0A061EYX3 | 42.5 | 198 | >TcKPI  MKTALFLALSFLLWGSTGATDGLDPVLDISGEELRTGTDYYILPVIRGGGGGLTLASTGNETCPLDVVQEQLEVSNGLPLTFSPVNITKGVVRVSTDLNIKFSAATICVQSTVWKLDNDEATQKMVVTTGGVEGNPGIETLSNWFKIERYEDDYKLVFCPGVCDFCRPVCGDLGVFIDAGIRRLALSDVPLKVMFKRA |
| 18 | Citrus clementina KPILP | *Rutaceae* | V4VP66 | 44.1 | 206 | >CcKPI  MRSTLVLPSLILLFAFTATPLPVKGNAQPDPVLDIAGKQLRAGSKYYILPVTKGRGGGLTLAGRGNNKTCPLDVVQEQHSFKNGLPVTFSPVNPKKGVVRESTDLNIKFDAATSCAQSTVWKLDNFDAAFGQWLVTTGGVEGNPGPRTMRNWFKIEKFFGDYKLVYCPSVCNFCRGLCRDVGIFINGGVRRLALSDIPFKVVFKKV |
| 19 | *Coffea canephora* KPILP | *Rubiaceae* | A0A068VJP3 | 41.9 | 204 | >CcaKPI  MKETLLPFFLSFLLFTSSPSSAARPASNAVRDIDGKEVIVGAHYYILPVIRGRGGGLTLGSADNETCPLDVIQEQFEVKRGLPLTFTPVNTTKGVVPVSTDLNIKFFAATICVQSTVWKLEFDADISQYVIVSGGIEGNPGRETISNWFKIEKYDQDYKLVYCPTVCNFCKVICRDVGIFVQNGRRRLALSDVPFRVVVKKAEY |
| 20 | Daucus carota subsp. Sativus  KPILP | *Apiaceae* | A0A166BVG9 | 42.0 | 201 | >DcKPI  MKKIRLYIPFLLVALSTCSLAAADASPDPVRDMDGDILRPGVDYYILPGVRGMGGGVTLGSTRNESCPLDVVQETFETDNGNLPLSFTMVDPKKGVIRESTDLNVEFNGVTICIQSLVWKLDNYDGEYVVSTRGVKGNPGAETLESWFKIEKYSNNYKFVYCPTVCDFCKPVCGDIGISIKDGFRRLVLSDQPFMVMFLKV |
| 21 | Glycine soja KPILP | *Fabaceae* | A0A0B2NRN9 | 37.1 | 203 | >GsKPI  MKVSPLAFSILFLSFTIELFIGIASAAQEPVLDTSGQKLRTGVKYYILPVFRGRGGGLTVSSSGNNTCPLFVVQEKLEVSKGTPVTFTPYNAESGVILTSTDLNIKSYVKSTTCDKPPVWKLLKVLTGVWFLSTGGVEGNPGVNTVVNWFKIEKAEKDYVLSFCPSFAQTLCRELGLYVGDDGNKHLSLSDKVPSFRVIFKRA |
| 22 | *Phaseolus vulgaris* KPILP |  | V7C6J1 | 35.1 | 205 | >PvKPI  MKASLLPFSILFFAFTIQLFIGIAVAAPEPVVDTSGQKLRTGVKYYILPVFRGRGGGLTVSSSGNNTCPLFVVQEKPEVLNGTPVTFTPYNAKSGVILTSTDLNIKSYGTTTSCDKPPVWKLLKVLTGVWFLSTGGVEGNPGIDTIVNWFKIEKAEKDYVISFCPSVCKCQTLCRELGLYVGDDGNKHLSLSDKVPSFRVMFKRA |
| 23 | Medicago truncatula KPILP |  | A0A072V908 | 39.2 | 198 | >MtKPI  MSTTLIKITSLSLMLCLFMSIKTLAQSENEKILDTKGHPLERGKEYYIKPAITDSGGRFTLIDRNGSCPLYVGQENTDLGKGLPVIFTPFAKEDKVIKDSRDFKVKFSASSICVQSTEWKLGDRDTKSGRRVIIAGSDGSYFRIVKAEFEGVYNIRFCPTDTCSFCRFDCGFVGGLRENGKILLALDGGVLPVVFVRA |
| 24 | *Trifolium repens* KPI-1 |  | U5NHH5 | 32.9 | 218 | >TrKPI-1  MKHVSSLTLSILFFVSITNLSLAFSNEDVEQVLDINGNAIFPGGEYYILPALRGPGGGGVRIGKTGDLKCPVTVLQDRREVKNGLPVKFTIPDISTGIIFTGTPVEIEFFKKPNCAKSSKWLVFVDNVVKKACVGIGGPENYPGVQTLSGTFNIHKHESGFGYKLGFCIKGSPTCLDIGRYDNDEAGKRLNLTEHESYHVIFVDAASHEADQYIKSVV |
| 25 | *Trifolium repens* KPI-2 |  | U5NHI1 | 30.9 | 220 | >TrKPI-2  MKPMLSLIFSFFLFILITNLSLALSNEAVEQVLDSLGNPIFPGGKYYIFPVSHDETYGGGLRLAKTGDSKCEVTALQDDNIVTESIPVKFSIPGISPGIIFTGTPIEIEFTKKPSCVESSKWLIFVDDVIQKACVGIGGPENYPHFKTLNDGRFYIEKHESGFGYKLGYCVKDSPTCLDIGRSGNVTEEGGFRLNLTHQVAYAVEFIDVTPFEARIKSVV |
| 26 | *Trifolium repens* KPI-4 |  | U5NEB5 | 28.0 | 210 | >TrKPI-4  MKPTMLTTLSLLLFALTTYFPLAFSSNEQLADLNGNPIFYSTHFYIMPSIFGAAGGGLKLGETGKLTCPLTVLQDYSEVINGLQLKFTPPGEIFVDLISTDQPLKGIEFVEKPECAESSKWVVVEDDDFPRPYVGIGGIEDNKGERIINGSFKIVKHGFGYKIVFCPRFTAPPGLCFDIGRHDDENGRRLILTENDPFEIVFVIPRRSVA  >TrKPI-4 ANRF  MVLDTRLCFVLDSLHHLVFVLILEGMMMRMEGVSSSLKMILLKLSL |
| 27 | Glycine max  KPI  (Gene KTI3) |  | P01070 | 29.17 | 216 | >GmKPI3  MKSTIFFLFLFCAFTTSYLPSAIADFVLDNEGNPLENGGTYYILSDITAFGGIRAAPTGNERCPLTVVQSRNELDKGIGTIISSPYRIRFIAEGHPLSLKFDSFAVIMLCVGIPTEWSVVEDLPEGPAVKIGENKDAMDGWFRLERVSDDEFNNYKLVFCPQQAEDDKCGDIGISIDHDDGTRRLVVSKNKPLVVQFQKLDKESLAKKNHGLSRSE |
| 28 | *Brassica oleracea* KPILP | *Brassicaceae* | A0A0D3DQ36 | 36.8 | 195 | >BoKPI  MSSLLYTFLLLAVFTSHRGATTEAAVEPVTDIYGQRLRTGTKYYILPVVRGRGGGLTMSKAENKTCPKSVIQERFEVSNGMPLTFSPSDKSKVIGVSTDLNFKFSATSIWNLDNVDEKTNQWFIGTCGVEGNPGRTTVGNWFKIDKYEDHYKILFCPSVCDICRVMCRDVGVFVQDGIRRLVLSDVPLKVMFKRA |
| 29 | *Brassica napus*  KPILP |  | A0A078D3D0 | 36.8 | 195 | >BnKPI  MSSLLCIFLLLAVFISHRGATTEAAVEPVTDIYGQRLRAGTKYYILPVVRGRGGGLTMSKAENKTCPKSVIQERFEVSNGMPLTFSPSDKSKVIRVSTDLNFKFSATSIWNLDNVDEKTNQWFIGTCGVEGNPGRTTVGNWFKIDKYEDHYKILFCPSVCDICRVMCRDVGVFVQDGIRRLVLSDVPLKVMFKRA |
| 30 | *Arabidopsis thaliana*  KPI-1 |  | A0A178W1G5 | 33.7 | 196 | >AtKPI-1  MSSLFYVFLLLAVFISHRGAITEAAVEPVKDITGKPLLTGFNYYILPVIRGRGGGLTMSNLKTETCPTSVIQDRFEVSQGLPVKFSPYDKSRIIPVSTDVNIKFSPTSIWELANFDETTKQWFISTCGVEGNPGQKTVGNWFKIDKFEKDYKIRFCPTVCNFCKVICRDVGVFVQDGKRRLALSDVPLKVMFKRAY |
| 31 | *Arabidopsis thaliana*  KPI-2 |  | Q8RXD5 | 33.3 | 205 | >AtKPI-2  MTKTTKTMNPKFYLVLALTAVLASNAYGAVVDIDGNAMFHESYYVLPVIRGRGGGLTLAGRGGQPCPYDIVQESSEVDEGIPVKFSNWRLKVAFVPESQNLNIETDVGATICIQSTYWRVGEFDHERKQYFVVAGPKPEGFGQDSLKSFFKIEKSGEDAYKFVFCPRTCDSGNPKCSDVGIFIDELGVRRLALSDKPFLVMFKKANVTEVSSKTM |
| 32 | Cucumis sativus  KPILP | *Cucurbitaceae* | A0A0A0LT81 | 34.5 | 195 | >CsKPI  MRNFALLCFLFIVIASSEVRFCRADASPDAVLDTDGKKLRAGDQYYILSVYSRNSGGLSIGGIYGYEKCPINILPESYDYLHGLPATFSPINPKKGVVRVSTDLNIQFEANTRCGISTVWKVGKFDEYLKQYFVTMGGMKGNPGRETIENWFKVEKYGKNYKLVYCPTVCKYCKVVCKDVGLFYKNGRRVIALNDAPFPVMFKKV |
| 33 | Hevea brasiliensis KPI | *Euphorbiaceae* | A0A0X7YK90 | 36.5 | 196 | >HbKPI  MLKLIGSLSFVWLLMAMSTVAQTPAVLDTNGQPLRSGVEYYVLPAATDTAGGLTLVNRTDSCPLYVGQEPLSTVVSQGLRVTFTPFAAQNDGIIRESREFSVAFSAVSICGQSTAWRVGEEDAETSRRFIVTGGEQSYFRIDNNGGLYELVWCPGESCTAPNCGRPRCGSAGILIENGKRLLALDGSAFPFRFRRA  >HbKPI ANRF  MEVSMSWYGALVSPALPLIAAGQDVVLLVF |
| 34 | *Ricinus communis* KPI |  | B9SIQ2 | 35.7 | 194 | >RcKPI  MVRSIGVSLGLACLLMAVSTIAQTPAVLDSSGQPLTSGVEYYVLPAATDTAGGLTLVNRTGSCPFYVGQEPLPTVAKTGFPVIFTPLAAGESIIREGMDFRVAFSAVTNCVQSTTWSIGDEDAETSRRFIVTGGEEDYFRIDKNEGLYNLGWCPNCNSINCPRPRCGFAGILIENGNRLLALDGAAFPFQFRRA |
| 35 | *Beta vulgaris*KPILP | *Amaranthaceae* | A0A0J8EV86 | 35.3 | 211 | >BtKPI  MTPHSILSILTILLFLLIAPLSTTTAAAKTTTVLDINGRPLKTDSTYYILPVSRGRGGGLAMAPKNATESCPLYVAQENHEVSNGLPLKFFPTNPNDHDKIPLGYDVNIVFDAATICVQPTGWMLAFDEASGTRYVGIGGTIGNPGVDTLSNWFAIEKAGSGLYDYKIRFCPAVCIFCTVMCGDVGVFIGEGGTRFLALTDRPLLVRFKKA |
| 36 | Spinacia oleracea  KPILP |  | A0A0K9QKP3 | 36.5 | 208 | >SoKPI  MTNFILSAATFLFFLLSSPPSTTAAAKITPVFDINGRPLQTGSTYYILPVIRGRGGGLTMTPINTTQSCPLYVAQENHEVSNGLPLKIYPVNPNEKRVPLGGDVNIVFDAASICVQSTGWMLAFDEATGKQHVATGGTIGNPGVDTLSNWFRIEKAGSGMYDYKVSFCPGVCIFCTVMCGDVGVFIGEDGTRFLGLTDRPLLVKFKKA |
| 37 | *Richadella dulcifica/ Synsepalum dulcificum* miraculin | *Sapotaceae* | P13087 | 34.5 | 220 | >MIRA_SINDU  MKELTMLSLSFFFVSALLAAAANPLLSAADSAPNPVLDIDGEKLRTGTNYYIVPVLRDHGGGLTVSATTPNGTFVCPPRVVQTRKEVDHDRPLAFFPENPKEDVVRVSTDLNINFSAFMPCRWTSSTVWRLDKYDESTGQYFVTIGGVKGNPGPETISSWFKIEEFCGSGFYKLVFCPTVCGSCKVKCGDVGIYIDQKGRRRLALSDKPFAFEFNKTVYF |
| 38 | Triticum aestivum KPILP | *Poaceae* | A0A1D5TYI6 | 32.7 | 228 | >TaKPI  MSSRRVGLLLLSLLATTLTCSADPPPVHDTDGNELRTDANYYVLPANRAHGGGLTMAPGHGRRCPLFVSQEADGQRDGLPVRIAPHGGAPSDKIIRLSTDVRISFRAYTTCVQSTEWHIDSELVSGRRHVITGPVRDPSPSGRENAFRIEKYSGAEVHEYKLMACGDSCQDLGVFRDLKGGAWFLGATEPYHVVVFKKAGRNSVLFLSLLENKCGAVLSSLRRRFRET |

*was retrieved from the Sol genomics network (<http://solgenomics.net/>)

**was predicted by <http://www.cbs.dtu.dk/services/TMHMM-2.0/>

*** database at the Dana-Farber Cancer Institute (DFCI, http://compbio.dfci.harvard.edu/tgi/).
