# Supplementary material for: An Alternative Nested Reading Frame May Participate in the Stress-Dependent Expression of a Plant Gene
Source: Front Plant Sci. 2017 Dec 19;8:2137. doi: 10.3389/fpls.2017.02137 (PMC5742262; doi:10.3389/fpls.2017.02137)
Supplement: Table S4 — KPILP matryoshka genes with embedded ANRFs. [file Table4.DOC]

Table S4. *KPILP* matryoshka genes with embedded ANRFs

| # | Plant and encoded protein name | EMBL ID | Nucleotide sequence* |
| --- | --- | --- | --- |
| 1 | *Nicotiana benthamiana* KPILP  (NbKPILP) | FN687760 | ATGCATTAACCATATCCTTAACA**ATG**AAGATCATATCAAGGATTTTATTGCTTTCTTGCCTCCTATTTTTAACCTTATTCCAGGTAAAAGCTGAACCAGTTCTTGATACTAATAAACAAGAAATCCGTCCAGGTTACACCTACTATATTTTGCCGGCAACCACCGCAAACGGCGGTGGCCTAACGCTAGCGAAAGGCGAAAACGGGAGCTGCCCGCTCGACGTTTTTCAAGCAAAAAATGTTCAGAGCACAGGCCTTCCTTTAAAATTCTTAATGGTGAATTCAAGTGCAGGGCTAGTAATTGACGAAAATGAAGACATAAATATAAAATTTGCAGCACCAAGGTACGTGTCGATTTGCAATAAATCCACTGTTT**GGAAAATTGAAGATGGGTTTGTGAG**CACTGGCGGAATTAAGGGTGGGTCAGAAA**ATG**GGACGGCCACAAGTTTGTTTACGATTCAGAAATATGAAGATGTCTATGCGTTACAGTATTGTCCAAGAGCTACAGGGTGTTCTTTTATTTGCCCCAGATTGTTGTGTGGGTATATTGGTATTTCACCTGCAGCTAATGGATCGAGGCGTTTGGCTGTGAATCGTCCAGTTTTCAAGATTGTGTTCAAGAAGGTTTAA |
| 2 | *Nicotiana glutinosa* KPILP  (NgKPILP) | AF208022 | CTGCAGGTAAAA**ATG**AAGATCATATCAAGGATTTTATTGCTTTCTTGCCTTCTATTTTTAACCTTATTCCAGGTAAAAACTGAACCAGTTCTTGATACTAATAAACAAGAAATCCGTCCAGGTTACACCTACTACATTTTGCCGGCAACCACCGGCGCCAACGGCGGTGGCCTAACGCTAGCGAAAGGCGAAAACGGGAGCTGCCCGCTCGACGTTTTTCAAGCACAAAATTCACAGAGCAGAGGCCTCCCTTTAAAATTCTTAATGGTGAATTCAAGTGCAGGACTAGTAATTGACGAAAATGAAGACATAAATATCAAATTTGCAGCACCAAGGTACGTGTCCATTTGCAATAAATCCACTGTTT**GGAAAATTGAAGACGGG**TTTGTGACCACTGGCGGAATTAAGGGTGGGTCTGAAA**ATG**GCACGGCCACAAGTTTGTTTACGATTCAGAAGTATGAAGATGCCTATGCGTTGCAGTATTGCCCAAGAGCTGCAGGGTGTTCTTTTATTTGCCCCAGATTGTTGTGTGGGTATATTGGTATTGCACCTGCAGCTAATGGGTCGAGGCGTTTGGCTGTAAATCGTCCAATTTTCAAGATTGAGTTCAAGAAGGCCTCAGATACAGAAGTGAATTTTTCACAATTTGATATTTATAATAGATGTGGATTTTCTAATACTTATATGAACAAAAGTTATTAG |
| 3 | *Solanum tuberosum* KPILP  (StKPILP) | XP_006353918 | GCAGAAAATATAATCATACTATTGATCAAT**ATG**AAGATTTTATTACTTTCTTGCTTCCTCTATTTTACCTTATTTCAAATCATAAAATCCGAACCGGTTCTCGATACTAGTAATGAACAAGTCCGTCCGGGGTACACCTACTACATATTGCCCGCTTCCGCGGGCAATGGTGGTGGCCTAACGCTAGCCAAAGGCGAAAACGGGAGCTGCCCCCTCGACGTTTTTCAAGCACGAAATTCTCAAAGTGTAGGCATCCCGTTGAAATTCTTGATGGTGAATTCTAGCGCGGGCTTAGTAATCGATGAAAATGAGGACATAAATATAAAATTCGCAGCGCGAAGGTACGTATCGATTTGTAACGTATCAACTGTTT**GGAAAATCGAAGATGGGA**TTGTGACTACTGGTGGAATTAAGGGTGGATCGGAAA**ATG**GCACATCTACAAGTTTATTTACGATTCAGAAATATGAAGATGCTTATGCTTTACAATATTGTCCTAGAGCTACAGGTTGTTCTTTTATTTGTCCAAGATTGTTGTGTGGGTATATTGGTATTTTACCAGCTGAAAATGGATCGAGGCATTTGGCTGTGAACCGGCCGGTTTTCAAGATTGTGTTCAGGAAGGCATAA |
| 4 | *Solanum lycopersicum* KPILP  (SlKPILP) | XM_004235444 | GTAAACAATTTCCCCATTAATAAAAACAACACTAATT**ATG**AAGATTTTATTACTTTCTTGCTTCCTCTACTTTACCTTATTTCAAACAATAAAATCCGAACCGGTTCTCGATACTAATAATGAACAAGTCCGTCCGGGGTACACCTACTACATATTGCCCGCGGCCTCCGCGGGCAGTGGTGGTGGCCTAACGCTAGCCAAAGGCGAAAACGGGAGCTGCCCCCTCGATGTTTTTCAAGCCCGAAATTCTCAAAGTTTAGGCATCCCGTTGAAATTCTTGATGGTGAATTCTAGCGCGGGTTTAGTAATCGATGAAAATGAAGACATAAATATAAAATTCGCAGCGCAGAGGTACGTATCGATTTGTAATGTATCGACTGTTT**GGAAAATTGAAGATGGGATTGTGA**CTACTGGTGGAATTAAGGGTGGATCGGAAA**ATGG**CACGTCTACGAGTTTATTTACGATTCAGAAATATGAAGATGCTTATGCTTTACAATATTGTCCTAGAGCTACAGGGTGTTCTTTTATTTGTCCAAGATTGCTGTGTGGGTATATTGGTATTTTAACAGCGGAAAATGGATCGAGGCATTTGGCTGTGAACCGGCCGGTTTTCAAGATTGTGTTCAGGAAGGCATAA |
| 5 | *Trifolium repens* KPI4  (TrKPI4) | KF022201.1 | ACCAATACACACATTCATTGCTTAACAACC**ATG**AAACCTACAATGCTTACCACCCTTTCTTTACTCCTCTTTGCCTTAACCACCTACTTTCCATTAGCTTTTAGTTCTAACGAACAACTAGCAGACTTGAATGGAAACCCCATCTTTTATTCTACTCATTTCTATATTATGCCATCTATCTTTGGAGCTGCAGGTGGTGGACTCAAGCTTGGTGAAACTGGAAAATTGACATGTCCACTTACTGTACTTCAAGATTATTCTGAAGTTATCAATGGTCTGCAACTAAAATTTACCCCTCCAGGTGAAATTTTCGTTGATTTGATAAGTACAGACCAACCACTGAAAGGTATTGAATTTGTAGAGAAGCCAGAGTGTGCTGAATCCTCCAAGTGGGTGGTGGTCGAAGACGATGATTTCCCTCGACCATATGTCGGAATTGGTGGTATT**GAAGACAATAAAGGTGAGAGGA**TCATAAATGGTAGCTTTAAAATTGTGA**A**AC**ATGG**TTTTGGATACAAGATTGTGTTTTGTCCTCGATTCACTGCACCACCTGGTCTTTGTTTTGATATTGGAAGGCATGATGATGAGAATGGAAGGCGTCTCATCCTCACTGAAAATGATCCTTTTGAAATTGTCTTTGTGATTCCTAGAAGATCTGTTGCTTGA |
| 6 | Hevea brasiliensis KPI  (HbKPI) | EM_PL:KM979450 | GTTACTTTAACCTTCTTTAGCGACC**ATG**TTGAAATTGATTGGAAGCTTAAGCTTCGTATGGCTTCTGATGGCCATGTCCACTGTGGCTCAAACCCCAGCAGTGCTAGACACCAACGGCCAGCCTCTTAGAAGCGGCGTAGAATACTACGTGCTTCCAGCTGCAACTGATACCGCTGGCGGTTTAACTCTGGTGAATCGCACTGACTCCTGCCCATTATACGTTGGTCAGGAACCTCTCTCAACCGTTGTCTCACAAGGCCTTCGAGTCACCTTCACGCCATTTGCTGCCCAAAACGACGGTATCATCAGGGAGAGCAGGGAGTTTAGCGTTGCATTTTCAGCTGTCTCCATCTGTGGCCAATCCACAGCAT**GGAGGGTAGGTGAGGAGGA**TGCAGAGACTTCAAGGAGATTTATAGTGACA**GGAGGAGAGCAGAG**TTACTTCAGGATTGACA**A**CA**ATGG**AGGTCTCTATGAGTTGGTATGGTGCCCTGGTGAGTCCTGCACTGCCCCTAATTGCGGCAGGCCAAGATGTGGTTCTGCTGGTATTTTGATTGAGAACGGGAAGAGATTGTTGGCCTTGGATGGCTCTGCTTTTCCTTTCCGATTTAGGAGGGCTTAG |
| 7 | *Arabidopsis thaliana*  KPI  (AtKPI) | At1g73260 | TCAACCCATATCTTCTTCATCCACAAATAACCATTGGCTATAATT**ATG**ACAAAAACTACCAAAACCATGAATCCTAAGTTTTACTTGGTTCTTGCCTTAACCGCGGTTCTGGCCTCAAACGCATATGGTGCGGTTGTAGACATCGATGGAAACGCCATGTTCCACGAAAGTTACTACGTTCTCCCTGTCATCCGTGGCCGAGGCGGAGGCCTGACTCTAGCAGGCCGCGGTGGGCAGCCATGTCCTTACGATATCGTGCAGGAATCTTCAGAAGTTGATGAGGGCATTCCCGTAAAATTCTCAAACTGGAGGCTTAAGGTTGCGTTCGTTCCCGAATCACAGAACCTCAACATCGAAACAGACGTCGGAGCCACGATCTGCATCCAGTCAACCTACTGGCGGGTCGGTGAGTTTGACCACGAGAGGAGGCAGTACTTCGTGGTTGCTGGTCCAAAGCCAGAAGGGTTCGGACAAGATTCGTTGAAGAGTTTCTTCAAGATCGAGAAATCTGGAGAGGATGCTTACAAGTTTGTGTTCTGTCCTCGGACTTGCGACTCTGGCAATCCAAAATGCAGCGATGTCGGGATATTCATAGATGAACTTGGCGTTCGTCGTTTGGCTTTAAGCGATAAGCCGTTCCTTGGTTATGTTCAAAAAAGCTAA |

*Initiating and terminating codons are underlined. PP blocks and ANRFs are highlighted in underlined and bolded red and yellow text, respectively.
